# The great live and move challenge and the promotion of physical activity in children: results from a two-school-year cluster-randomized trial

**DOI:** 10.1186/s12966-025-01849-x

**Published:** 2025-12-01

**Authors:** Mathieu Gourlan, Céline Lambert, Bruno Fregeac, Lucile Mora, Florian Jeanleboeuf, Adrien Minotte, Olivier Coste, Bruno Pereira, Florence Cousson-Gélie

**Affiliations:** 1 Epidaure-Prevention Department of the Montpellier Cancer Institute, Parc Euromédecine, 208 Avenue des Apothicaires, Montpellier cedex 5, 34298 France; 2 Univ. Montpellier, EPSYLON EA 4556, Route de Mende, Montpellier cedex 5, 34199 France; 3 Biostatistics Unit, CHU Clermont-Ferrand, DRCI, Villa annexe IFSI, 58 rue Montalembert, Clermont Ferrand, 63003 France; 4 Academic Resource Center of Hérault Dedicated to Health Promotion, 208 Avenue des Apothicaires, Montpellier cedex 5, 34298 France; 5 Dahlir, 8 impasse du viaduc, Brives-Charensac, 43700 France; 6 Direction de Région Académique à la Jeunesse, à l’Engagement et aux Sports Occitanie, 3 avenue Charles Flahault, Montpellier Cedex 5, 34094 France

**Keywords:** Physical activity, Primary school children, Cluster-randomized controlled trial, Primary prevention, Theory of planned behavior, Intention-behavior gap

## Abstract

**Background:**

Population surveys indicate that a large proportion of children worldwide do not meet the recommended 60 min of moderate-to-vigorous physical activity (PA) daily. This study aimed to assess the impact of the Great Live and Move Challenge (GLMC), a theory of planned behavior (TPB)-based intervention, on PA and TPB variables in French primary school children over two school years. Secondary objectives included assessing whether TPB variables mediated the GLMC’s impact on PA and whether the GLMC impacted the strength of the link between TPB variables.

**Methods:**

A cluster-randomized controlled trial was conducted over 16 months. One hundred primary schools were randomized into an intervention or control group. A total of 2723 children aged 7–11 years (mean age: 9.1; 49.7% boys) were included (*n* intervention = 1420, *n* control = 1303). The GLMC, based on TPB, comprised a motivational phase (targeting attitudes, subjective norms, and perceived behavioral control) and a volitional phase (notably focusing on the intentions-PA link). The intervention involved teachers, parents, and community stakeholders. The primary outcome was the change in the proportion of children meeting PA guidelines after 16 months. Secondary outcomes included changes in mean daily PA and TPB variables. Assessments occurred at baseline, 4, 12, and 16 months. Data were analyzed using mixed models and path analyses.

**Results:**

The “time × group” interaction indicated that, compared with the control group, the intervention group had a significantly higher proportion of children meeting international PA guidelines after 16 months (OR = 3.38, 95% CI = 2.50 to 4.56, *P* < 0.001). TPB variables did not significantly mediate the impact of the GLMC on mean daily PA at 16 months. The path coefficient between intentions and mean daily PA was significantly higher in the intervention group than in the control group at 4 (CR = 2.45; β = 0.22 vs. 0.13) and 16 months (CR = 2.87; β = 0.24 vs. 0.14).

**Conclusions:**

The GLMC increased the proportion of children meeting PA guidelines over 16 months, and may help bridge the intentions-PA gap. The absence of mediation by TPB variables highlights the need to investigate other psychosocial mechanisms to better understand how the GLMC promotes PA in children.

**Trial registration:**

International Standard Randomized Controlled Trial Number (ISRCTN) Registry: 61,116,221 (retrospectively registered).

**Supplementary Information:**

The online version contains supplementary material available at 10.1186/s12966-025-01849-x.

## Background

Physical activity (PA) in children provides various health benefits, including reduced obesity, improved fitness, motor skills, cognitive functioning, psychological well-being, and positive social interactions [1–4]. Engagement in PA is crucial for children aged 5–12 to establish lifelong habits [5, 6]. International guidelines recommend a minimum of 60 min of moderate-to-vigorous daily PA for children [5]. However, population surveys have reported insufficient PA levels, with fewer than 40% of children meeting international guidelines in the United States [7], Australia [8], or in European countries [9], including France [10]. In this context, enhancing PA in children is recognized as a public health priority [11]. Accordingly, the present study focuses on French primary school children aged 7–11 years.

To date, interventions targeting children’s PA levels have shown negligible or non-significant effects, as reported in meta-analyses by Metcalf et al. [12] (*d* = 0.12), Love et al. [13] (*d* = 0.02), Rodrigo-Sanjoaquín et al. [14] (*g* = 0.07), and Neil-Sztramko et al. [15] (Mean difference [in minutes of daily PA] = 1.01). A lack of understanding of intervention mechanisms has been suggested as a barrier to developing effective PA programs [16]. Since many theories successfully explain health behaviors like PA [17], applying theory to develop and assess PA interventions in children is recommended [18].

The Theory of Planned Behavior (TPB) is a widely used model in health behavior interventions [19, 20]. According to this model [19], behavioral intentions (an individual’s motivation to act) are the most immediate antecedent of behavior adoption. Intentions, in turn, are determined by attitudes (beliefs that make a more or less favorable or unfavorable opinion toward behavior), perceived subjective norms (beliefs of how significant others display behavior and beliefs of perceived social pressure from these significant others to display behavior), and perceived behavioral control (perceived capacity from an evaluation of resources and opportunities to display behavior). Perceived behavioral control is also hypothesized to predict behavior directly. Numerous studies have supported the TPB’s validity in explaining children’s PA behavior [21, 22].

Previous TPB-based interventions have targeted adolescents [23–25] or primary school children [26, 27]. In most randomized controlled trials, TPB-based interventions significantly impacted PA compared to a control group [24–27]. In line with the principle of implementing theory-based interventions [28, 29], TPB-based interventions should also report whether the TPB model mediates the intervention’s impact on PA. Some studies reported that, compared to the control group, TPB-based interventions significantly impacted the TPB-determinant(s) targeted to promote PA in youth [23, 24, 27]. However, only Hill et al. [24] confirmed that perceived behavioral control and intentions partially mediated the significant impact of their intervention on adolescents’ PA.

An important issue in TPB-based interventions is that a change in intentions due to a change in at least one determinant of intentions may only result in a modest behavior change [30]. This phenomenon is called the “intentions-behavior gap” [31]. For instance, a meta-analysis reported that PA interventions successful in increasing intentions in adults (*d* = 0.45) resulted in a lower impact on PA (*d* = 0.15) [32]. The efficacy of TPB-based interventions may be optimized by including content that helps individuals translate their intentions into behavior [20, 28, 33]. A promising strategy is to focus on actual behavioral control [30], which reflects individuals’ existing resources, opportunities, and skills to perform a behavior [34]. Implementing PA sessions during the intervention, once intentions have been developed, may allow children to experiment and develop their awareness and knowledge about their actual control toward PA practice [35]. Additionally, using implementation intentions, a self-regulatory planning strategy [36], is another promising means for bridging the intentions-behavior gap within TPB-based interventions [28, 33]. Implementation intentions are an if-then plan specifying when, where, and how an individual will instigate a goal-directed behavioral response [37]. Previous research has confirmed the efficacy of implementation intentions to promote PA, including in children [38, 39].

From a theoretical perspective, including content that facilitates the translation of intentions into actual PA can enhance the efficacy of TPB-based interventions by strengthening the statistical link between intentions and PA [40]. Many observational studies have quantified the intentions-PA gap [32, 41] or explored factors that moderate the link between intentions and PA [42], and some interventional studies have reported the increased impact on PA of interventions that include contents facilitating the translation of intentions into behavior [43, 44]. However, no research has examined whether an intervention could statistically strengthen the direct link between intentions and PA [45], including in children [46].

### The present study

The Great Live and Move Challenge (GLMC) is a TPB-based intervention to promote PA in French primary school children aged 7–11. The primary purpose of this study was to evaluate the 16-month effect (i.e., two school years) of the GLMC on PA and TPB variables. Children in the intervention group participated in two GLMC editions (one each school year). As previously recommended for TPB-based interventions [47], the GLMC included both “motivational” and “volitional” phases. Also, in line with general recommendations for theory-based interventions [29, 48], each content of the GLMC was explicitly linked to a theoretical variable (i.e., attitudes, subjective norms, perceived behavioral control) or to the translation of intentions into behavior (i.e., intentions-PA link) through the selection of specific behavior change techniques (BCTs) [49]. The motivational phase included BCTs targeting attitudes, subjective norms, and perceived behavioral control to increase intentions toward PA [28, 29]. The volitional phase included BCTs targeting perceived behavioral control to impact PA directly [47] and to translate increased intentions into actual PA practice (i.e., PA sessions, implementation intentions) [28, 33]. A pilot study reported a significant change in TPB variables and PA among GLMC participants over one school year [50]. However, the absence of a control group and a volitional phase in the GLMC limited conclusions about the efficacy of the intervention [50, 51]. The present study treated PA as both a dichotomous and continuous outcome variable. The dichotomous measure was the proportion of children meeting international guidelines of at least 60 min of daily PA [5], aligning with the GLMC’s public health objective [52]. The continuous measure was the mean daily duration of PA in minutes (i.e., mean daily PA), coherent with the behavior concretely emphasized in the GLMC intervention (i.e., engaging in more PA daily; see below). It was hypothesized that the proportion of children meeting international guidelines of at least 60 min of daily PA would increase by 15% in the intervention group compared to the control group (H_1a_) [52]. It was also hypothesized that children in the intervention group would report significantly higher mean daily minutes of PA, attitudes, subjective norms, perceived behavioral control, and intentions than those in the control group (H_1b_).

The secondary purpose was to determine whether TPB variables were involved in the impact of the GLMC. It was hypothesized that over the 16-month follow-up, the TPB model would mediate the impact of the two GLMC editions on mean daily PA in the intervention group compared to the control group (H_2a_). Additionally, the study explored how the link between TPB variables varied by randomization group. It was hypothesized that through the volitional phase of the GLMC, the link between intentions and mean daily PA would be significantly stronger in the intervention group compared to the control group after each of the two GLMC editions (H_2b_).

## Methods

### Design

The GLMC trial was a 16-month cluster-randomized controlled trial comparing an intervention group to a passive waiting list control group. A total of 2723 children from 201 classrooms taught by 201 teachers across 100 primary schools in three French departments (Aude, Gard, Hérault), southern France, participated in this study. This trial followed the Consolidated Standards of Reporting Trials (CONSORT) guidelines (see Additional file 1), and the study protocol is detailed elsewhere [52]. Data were collected at four time points over 16 months (i.e., two school years): January-February 2016 (pre-intervention of the first school year, baseline), May-June 2016 (post-intervention of the first school year, 4 months), January-February 2017 (pre-intervention of the second school year, 12 months), and May-June 2017 (post-intervention of the second school year, 16 months).

The study was approved by the French Ethics Committee “Sud Méditerranée I”, the French Advisory Committee on Information Processing in Material Research in the Field of Health (registration no. 15279) and the French Data Protection Authority (registration no. 1860542). The study is also registered in the International Standard Randomized Controlled Trial Number (ISRCTN) database (registration no. 61116221).

Of note, the variables and analyses related to the secondary objectives (H_2a_ and H_2b_) were refined compared to those specified in the registered protocol [52]. In addition to the TPB-based motivational variables and mean daily PA reported as secondary outcomes in the present study, other secondary outcomes were measured in the trial but are not presented here. These include children’s PA planning and perceived opportunities for PA, as well as parental outcomes, namely parental social support and parental involvement in shared family PA (see [52]). Parental outcomes are planned to be reported in a separate publication. In addition, although children’s planning and perceived opportunities for PA were listed in the protocol, we came to consider that the volitional phase of behavior change in the GLMC was mainly targeted through mechanisms such as actual behavioral control and implementation intentions (see above), which were embedded in the intervention but could not be directly measured [53]. Therefore, the current analyses related to the secondary objectives (H_2a_ and H_2b_) focused on the motivational core of the TPB (i.e., attitudes, subjective norms, perceived behavioral control, and intentions), along with mean daily PA. Finally, specifically regarding H_2b_ analyses, instead of conducting sequential mediation and moderation analyses with the PROCESS macro as planned [52, 54], we opted for a path analysis approach. This method allowed for estimating the TPB model as a whole across the 16-month follow-up, to test for differences in the paths between intervention and control groups, particularly the path from intentions to mean daily PA, and to appropriately handle missing data. Overall, despite these refinements, the present analyses address the core part of the secondary objectives specified in the protocol, namely the effects of the GLMC on TPB variables and the involvement of these variables in explaining the intervention’s impact on PA [52].

### Participants

Participants were children aged 7 to 11 (grades 2 to 5). All children from participating classrooms were eligible to participate. Written parental consent was obtained before baseline [52]. All children were also invited to take part in the accelerometer-based assessment, for which additional parental consent was requested. Exclusion criteria were: (i) parental refusal, (ii) medical contraindications to PA reported by parents and documented by a physician (e.g., cardiac disease, severe orthopedic limitations), (iii) inability to speak or read French, and (iv) absence at baseline. All children were verbally informed about the study at baseline, reminded of their right to decline participation, and provided oral assent before each time point.

### Procedures

Before randomization, educational advisers from the local academic authority contacted the principals of 1012 eligible primary schools and asked them to inform teachers from the relevant grades about the opportunity to participate in the GLMC trial. Teachers were then free to decide whether or not they would involve their class in the trial. Educational staff and teachers from primary schools could contact the trial coordinator for more information about the study. There was no cap on the number of participating teachers per school, which was nonetheless limited by the fact that the GLMC trial was only offered to teachers of the targeted school grades. Depending on school size, between one and seven teachers actually participated per school.

**Fig. 1:**
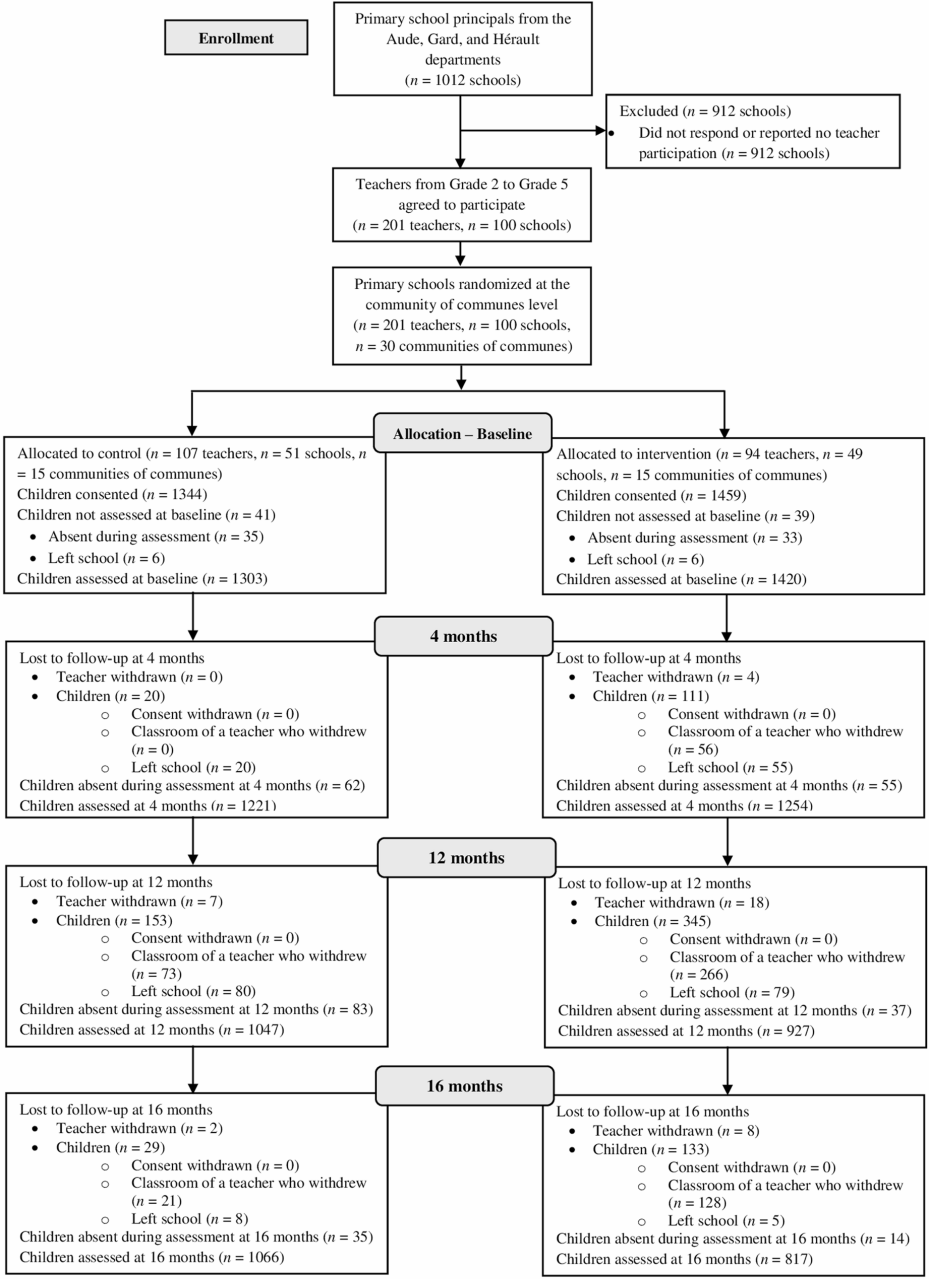
CONSORT flow diagram of participants throughout the study Note: Baseline, pre-intervention of first follow-up year; 4 months, post-intervention of first follow-up year; 12 months, pre-intervention of second follow-up year; 16 months, post-intervention of second follow-up year

In this trial, randomization was done at the level of communities of communes (French inter-municipal authorities grouping neighboring municipalities) [55], aiming to prevent contamination between intervention and control groups since the GLMC included weekend PA sessions for children and their families in the intervention group’s neighborhoods (see below). The community of communes, as the unit of randomization, corresponds to a local territorial unit encompassing several neighboring municipalities and their schools, responsible for coordinating some educational, cultural, and PA initiatives across municipalities. The communities of communes were stratified by the French department (Aude, Gard, Hérault) and residential environment (urban, rural) for randomization, as sociodemographic backgrounds and PA level could differ across these two contextual factors [56–59]. This randomization was performed prior to primary school recruitment [60], and responded to a specific request from the local academic authority to ensure that all educational advisers, who are the official contacts for primary schools within their respective jurisdictions, felt involved in the school recruitment process (see above). While blinded to group allocation, these advisers were informed that their territory had been randomized for participation in the research project, which facilitated their outreach to all primary school principals within their jurisdiction. An independent statistician generated cluster randomization of the 30 communities of communes, using a 1:1 allocation ratio within each stratum (*n* intervention = 15, *n* control = 15) (see Fig. 1). All 30 randomized communities of communes were represented by at least one participating primary school, ensuring that each cluster contributed data to the trial. The number of participating schools per community of communes ranged from 1 to 11 (mean = 3.3, SD = 2.7).

Children completed the questionnaire in their classroom for 45 min at each time point, supervised by their teacher and study assessors (two to three assessors according to school class size). To minimize potential comprehension difficulties, one assessor read each item aloud to the class, while at least one second assessor provided individual support if required (e.g., reformulating the item or providing examples). The GLMC staff (composed of a teacher, doctor, researchers, and professional health educators) led the preparation of the GLMC with the multiple local grass-roots partners in the intervention group (i.e., teachers, municipal officials, and policy stakeholders from community agencies and district councils). The study assessors were blinded to the group assignment (intervention vs. control) only at baseline. The GLMC staff and multiple local grass-roots partners could not be blinded to the group assignment prior to baseline measures (January- February 2016), as the preparation of the GLMC occurred during that time (see below) [52].

Given that the GLMC trial was conducted over two consecutive school years, particular attention and organizational efforts were devoted, in coordination with the local academic authority, to ensuring that children remained in a class continuing to participate in the GLMC within their school as they progressed to the next grade. Concretely, when a school had only one participating teacher (see above), efforts were made to ensure that children remained with the same teacher over both school years, as teachers may change the grade they teach from one year to the next. In schools with several participating teachers, organizational arrangements were made to ensure that children remained in a class led by a teacher involved in the trial, who could be the same as in the first school year. Children who moved out of the participating schools, most often between the first and second school years (i.e., between the 4- and 12-month time points), were not followed up and were considered lost to follow-up for subsequent assessments (see Fig. 1).

### Interventions

#### Great live and move challenge preparation and intervention

The Template for Intervention Description and Replication (TIDieR) Checklist was used to describe the intervention (see Additional file 2). Children in the intervention group benefited from the GLMC over two school years [52], delivered annually over three and a half months (January to mid-April). The intervention was designed to be implemented identically in both school years, with the same contents, timeline, and grass-roots partners involved in delivering the sessions, to promote consistency in how the intervention was delivered among participating children regardless of the year of implementation. It comprised two main steps each school year: (i) preparing the GLMC with local grass-roots partners over two months (January and February) and (ii) implementing the GLMC intervention for children over one month and a half (March to mid-April).

During GLMC preparation, teachers (*n* = 94) and municipal officials (*n* = 29) received role-specific intervention guides [52] and could contact GLMC staff for support (e.g., advice on sessions). Additionally, GLMC staff assisted policy stakeholders from community agencies and district councils (*n* = 17) in delivering PA sessions for children and families on weekends during the GLMC (see below).

The GLMC intervention, based on TPB, aimed to promote PA in children through seven modules (see [52] for a full description). In line with a core principle of theory-based interventions [29, 48], each module was designed to impact a TPB variable (attitudes, subjective norms, or perceived behavioral control). Some modules were also designed to strengthen the intentions-PA link [47]. The contents of each module were formalized via an existing taxonomy of BCTs [49]. The information related to the contents, the grass-roots partner(s) who implemented session(s), the TPB variable or process targeted, and the BCTs used for each module are summarized in Table 1. In line with existing recommendations [47], Modules 1 to 5 included BCTs targeting attitudes, subjective norms, and perceived behavioral control to promote children’s intentions toward PA in the “motivational phase” during the first two weeks of the GLMC. Subsequently, module 2, which included BCTs targeting perceived behavioral control, and modules 6 and 7, with BCTs targeting the intentions-PA link, formed the one-month “volitional phase” of the GLMC [47]. In concrete terms, a central aspect of the GLMC was encouraging children to cumulate “energy cubes” (equivalent to 15 min of continuous PA; see module 2 in Table 1) during the “volitional phase.” Children were informed that they were all part of a “GLMC community” with a shared goal of accumulating maximum “energy cubes” altogether. Another key content of the GLMC during the “volitional phase” was the implementation of PA sessions for children by teachers during school time, by municipal officials during extracurricular time, and for children and families by policy stakeholders during the weekends (see module 7 in Table 1). All PA sessions were provided free of charge.

**Table 1:** Summary of the modules of the great live and move challenge intervention

Phase (duration) and module of the GLMC	Main TPB variable or process targeted	Main BCT(s) used^a^ [scientific evidence^b^]	Summary of the contents of the module	Number of sessions (frequency)	Module implemented by
“Motivational Phase” (two weeks)					
Module 1: “What is PA?”	Subjective norms	Information about social and environmental consequences [61]	• Presentation of the notion of PA (e.g., sports activities, leisure times PA, lifestyle PA) • Presentation of the PA guidelines for children (i.e., 60 min of moderate-to-vigorous PA per day) • Presentation of the general principles of the GLMC with the video entitled “Presentation of the GLMC” (e.g., absence of any competitive aspect, GLMC calendar)	1 session	Teachers
Module 2: “Let’s count energy cubes!”	PBC	Self-monitoring of behavior [62]	• Distribution and presentation of the GLMC diary for children • Presentation of the “energy cube” notion: One “energy cube” equals 15 min of continuous PA. When practicing family PA, each child accumulates additional “energy cube(s)” for each family member who has practiced with him/her. For example, if a girl taking part in the GLMC takes a 15-minute bicycle ride with her mother and her brother, she accumulates 3 “energy cubes” • Exercises to learn how to correctly count the number of “energy cubes” (e.g., when practicing PA with and without family members, how to correctly complete the“energy cubes” counting table in the GLMC diary)	2 sessions (weekly sessions during the “motivational phase”)	Teachers
Module 3: “Let’s talk about why we should do PA?”	Attitudes	Information about health consequences, Information about social and environmental consequences, Information about emotional consequences [23]	• Presentation of the video “Let’s talk about PA – The advantages to practicing PA regularly” (e.g., for “being in good health”, “being in a good mood”, “having a great time with family and friends”) • Discussion with children about the personal benefits they perceive in practicing PA	1 session	Teachers
Module 4: “Let’s talk about how we should do PA?”	PBC	Instruction on how to perform a behavior [63]	• Presentation of the video entitled “Let’s talk about PA – How to practice PA regularly” (e.g., tips about the activities, times of the week to practice PA) • Each child identifies some combinations linking some new PA to do, where, when, and with whom to practice those activities	1 session	Teachers
Module 5: “Let’s encourage all children to do PA!”	Subjective norms	Information about health consequences, Information about social and environmental consequences, Information about emotional consequences [64]	• PowerPoint presentation to parents entitled “How to promote PA in our children?” including the benefits of shared family PA (e.g., for “having fun”, “strengthening family relationships”) and of supporting one’s child toward PA (i.e., for “helping children take responsibility for their health”)	1 session	Teachers, Municipal officials, Local policy stakeholders
• “Volitional phase” (four weeks)					
Module 2: “Let’s count energy cubes!”	PBC	Self-monitoring of behavior [62]	• Children are encouraged to record daily the number of “energy cubes” they have accumulated on their GLMC diary • A weekly update is made on the number of “energy cubes” cumulated by children in every classroom	4 sessions (weekly sessions during the “volitional phase”)	Teachers
Module 6: “Let’s make PA plans!”	Intentions-PA link (implementation intentions)	Action planning [39]	• Through an implementation intentions exercise, each child plans the number of “energy cubes” he/she will try to cumulate during the coming week during moments at school (e.g., recesses)	4 sessions (Weekly sessions during the “volitional phase”)	Teachers
Module 7: “Let’s practice PA altogether!”	Intentions-PA link (actual behavioral control)	Behavioral practice/rehearsal [35]	• In schools, teachers organize PA sessions (e.g., Olympiad) for children during school hours (in addition to PE lessons) • In recreation centers, municipal officials organize PA sessions for children during extracurricular time (e.g., treasure hunt) • In cities, local policy stakeholders organize weekend PA sessions designed for children and their families in one of the city’s neighborhoods (e.g., family hike, giant Zumba lesson)	At least one weekly PA session during the “volitional phase”	Teachers, Municipal officials, Local policy stakeholders

Table adapted from Cousson-Gélie et al. [52]

*Abbreviations BCT *behavior change technique, *GLMC* Great Live and Move Challenge, *PA* physical activity, *PBC* perceived behavioral control, *PE* physical education, *TPB* theory of planned behavior

^a^From the behavior change technique taxonomy V1 (BCTTV1) [49]

^b^Scientific evidence of the impact on the variable or link between variables targeted

#### Passive control group

During the protocol, children in the control group did not receive any school-based intervention to promote PA. However, in line with the French national curriculum [65], all children in the control group were expected to receive three hours of physical education per week, delivered by their teacher. After the protocol ended, teachers from the control group were invited to implement the GLMC at the beginning of the following school year.

### Measures

#### Outcome variables

The primary outcome was the change in the proportion of children meeting international guidelines of at least 60 min of daily PA after 16 months [52]. Self-reported PA level was assessed at each time point for all children using an adapted version of the self-administered Physical Activity Questionnaire for Children (PAQ-C) [66], which had been used in a previous study [22]. This questionnaire asked about PA duration in minutes in various contexts over the past seven days, including sports clubs, school recess, lunchtime, free time, and daily life. The mean daily PA minutes were calculated by summing weekly minutes across contexts and dividing by seven [67]. Children were classified as meeting (≥ 60 min/day) or not meeting (< 60 min/day) the international PA guidelines [5].

In addition to self-reported PA, a subsample of children was assessed using accelerometers to provide concurrent validity with the PAQ-C. In line with H_1a_, exploratory analyses were also conducted in this subsample to examine potential GLMC effects on the proportion of children meeting international PA guidelines, based on both accelerometer- and self-reported data. A total of 340 children had parental consent to participate in the accelerometer-based assessment (see Additional file 3). From this group, a random subsample (*n* = 232), already allocated to the intervention or control group as part of the main trial, was selected to wear an accelerometer (Triaxial GT3X+, LLC, Pensacola, Florida, USA) at each time point (n intervention = 111; n control = 121). Following recommendations [68, 69], children were asked to wear the accelerometer around their waist for seven consecutive days, during all waking hours (i.e., excluding sleep), and to remove it for water-based activities (e.g., swimming, bathing, showering). Data, sampled at 30 Hz and accumulated into 1-s epochs with Actilife software (V.6.13.3, LLC, Pensacola, FL, USA) [70], were analyzed with non-wear time defined as at least 60 consecutive minutes of zero counts, allowing for up to 2 min of counts between 0 and 100 only [71]. Additionally, only children with at least five valid days, defined as days with ≥ 480 min of wear time, including at least one weekend day, were included in the analyses [68, 69]. Accelerometry-based mean daily minutes of PA were calculated using the age-appropriate Evenson et al. (2008) cut-point of ≥ 2296 counts per minute [72, 73]. As with self-reported PA, children in the accelerometer-wearing subsample were classified as meeting or not meeting international PA guidelines.

#### Theory of planned behavior variables

At each time point, all children completed a TPB questionnaire measuring attitudes (6-item), subjective norms (8-item), perceived behavioral control (6-item), and intentions (3-item) to practice PA almost daily. In this questionnaire, used in a previous study [22], the wording of all items and the response options were amended according to previous TPB research involving children (e.g., [74, 75]). Children responded to all items using a Likert-type 4-point scale, scoring (i) no, not at all; (ii) no, not really; (iii) yes, maybe; and (iv) yes, for sure.

#### Sociodemographic variables

Children self-reported their age and gender at baseline.

### Statistical methods

#### Sample size

The intervention targeted an absolute 15% difference between the intervention and control group at 16 months in the change of proportion of children meeting international PA guidelines [52]. The required sample size was estimated at 4000 children with a two-sided 0.05 error, 80% statistical power, a mean of 25 children per classroom, and an intra-class correlation coefficient of 0.005 for the communes of a given community of communes [52].

#### Statistical analysis

Statistical analyses were performed using Stata software (version 16; StataCorp, College Station, TX, USA) for H_1a_ and H_1b_, and AMOS (version 21; SmallWaters Corp., Chicago, Illinois, USA) for H_2a_ and H_2b_. All tests were two-sided, with an alpha level of 0.05. Main analyses were conducted according to the intention-to-treat principle on the full analysis sample (*N* = 2723) after imputing missing data. Missing data were found to be random (Little’s test *P* < 0.001). Missing values were imputed for all analyses using the full information maximum likelihood procedure [76, 77]. To assess the robustness of the main findings, additional complete case analyses (*N* = 1680), defined as children with available data at baseline, 4-, 10-, and 16-month time points, were conducted as sensitivity analyses for H_1a_, H_1b_, H_2a_, and H_2b_.

Hypotheses H_1a_ and H_1b_ were studied with logistic or linear mixed models, with children and community of communes as random effects. Independent variables included the randomization group, the time (baseline, 4, 10, and 16 months), the interaction between the randomization group and the time, the children’s gender and age at baseline, and the baseline value of the dependent variable. A logarithmic transformation was applied to quantitative dependent variables to achieve normality if appropriate. Of note, although the intra-class correlation coefficient for PA was anticipated to be very low (0.005) in our protocol [52], we conducted an additional sensitivity analysis for H_1a_ on complete cases (*N* = 1680), including a random effect for school class (i.e., group of children sharing the same pedagogical and social environment with their teacher during a given school year), in addition to community of communes, to empirically confirm that clustering effects did not alter the conclusion related to the primary outcome of the study. This additional level was considered because children from the same school class share a common pedagogical and social environment (e.g., teaching practices, teacher-children interactions, and peer dynamics) that may create statistical dependence [50]. Results are expressed as odds ratio (OR) and 95% confidence interval (CI) for logistic regressions and as effect size (ES) and 95% CI for linear regressions. OR of < 1.50 was interpreted as very small or trivial, ≥ 1.50 was interpreted as small, ≥ 2.50 as medium, and ≥ 4.30 as large [78]. ES of < 0.10 was interpreted as very small or trivial, ≥ 0.10 as small, ≥ 0.30 as medium, and ≥ 0.50 as large [79].

**Fig. 2:**
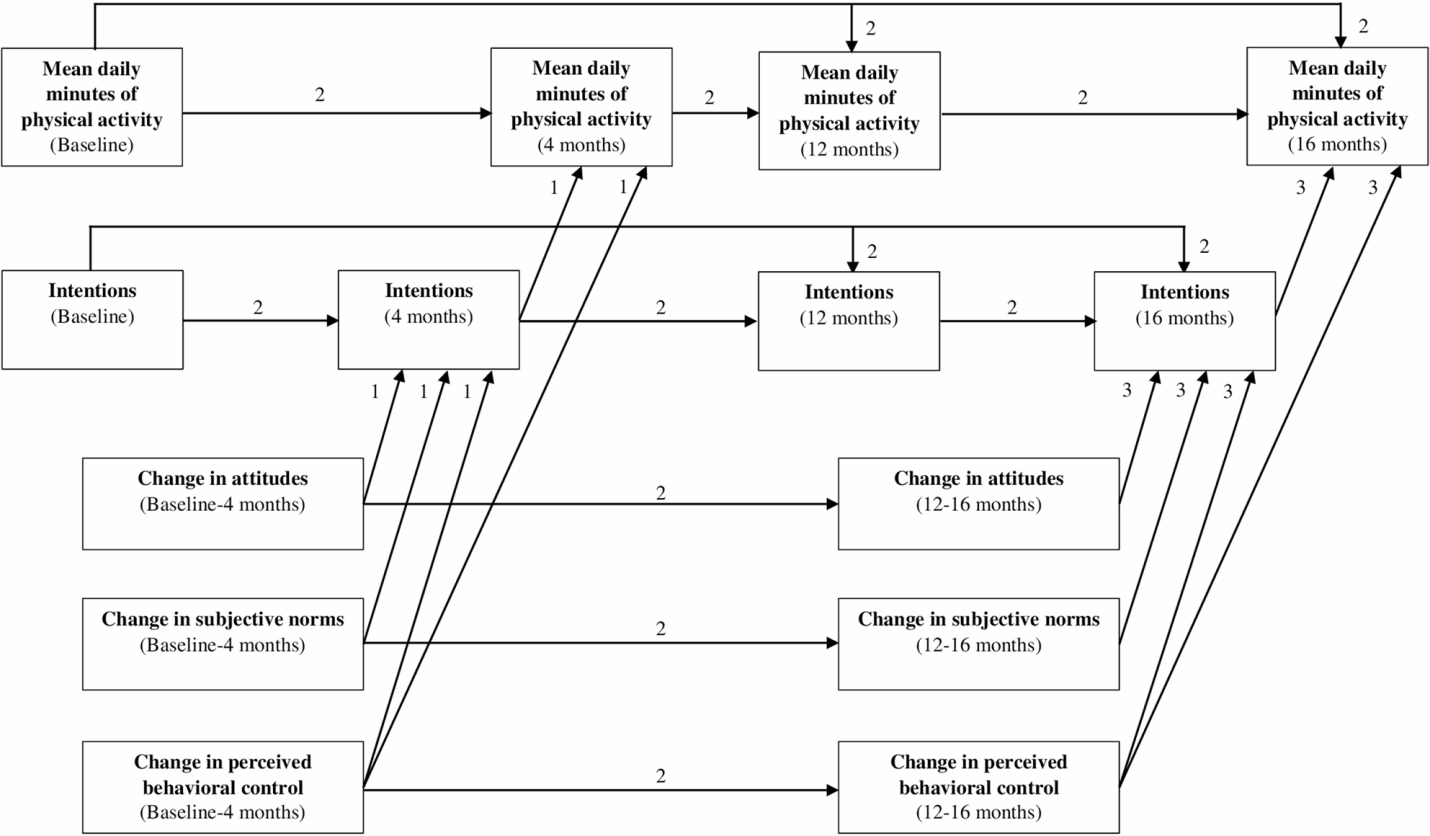
Presentation of the basic model used to explore the underlying mechanisms of the impact of the Great Live and Move Challenge on children’s mean daily physical activity Note: 1, paths for the theory of planned behavior variables between baseline and 4 months; 2, autoregressive paths; 3, paths for the theory of planned behavior variables between 12 and 16 months. Baseline, pre-intervention of the first school year; 4 months, post-intervention of the first school year; 12 months, pre-intervention of the second school year; 16 months, post-intervention of the second school year. Effects of gender and age on mean daily minutes of physical activity at baseline, 4 months, 12 months, and 16 months have been omitted for clarity, but standardized parameter estimates for the direct and indirect effects of each path can be in Table 6 and Additional file 12, respectively

Hypotheses H_2a_ and H_2b_ were tested through two path analysis models using a maximum likelihood estimator. The basic model, common to H_2a_ and H_2b_, is presented in Fig. 2 (see also Additional file 4 for a detailed presentation of the basic model). To evaluate whether the GLMC impacted mean daily PA through TPB variables (H_2a_), the randomization group was added as an antecedent of variables in the basic model (Fig. 2). To evaluate whether the link between TPB variables varied by randomization group (H_2b_), the basic model (Fig. 2) was tested separately for the intervention and control group subsamples. Model fit for H_2a_ (whole sample) and H_2b_ (intervention and control group subsamples) was assessed using the chi-square index (χ²), comparative fit index (CFI), Tucker-Lewis index (TLI), root mean square error of approximation (RMSEA), and standardized root mean square residual (SRMR). Thresholds for good fit were >0.90 for the CFI and TLI and < 0.08 for the RMSEA and SRMR [80]. For direct effects in the models, β of < 0.10 was interpreted as very small or trivial, ≥ 0.10 as small, ≥ 0.20 as medium, and ≥ 0.30 as large [81]. Specifically for H_2b_, the statistical difference in each path coefficient of the basic model between subsamples was assessed using the critical ratio (CR) statistic. A CR >1.96 indicates a significant difference between subsamples [82].

## Results

### Sample characteristics

Parental consent was obtained for 2803 of 4686 eligible children (59.8%) at baseline (*n* control = 1344; *n* intervention = 1459) (see Fig. 1). Eighty children were not assessed at baseline (absent or left school), yielding a total sample of 2723 children (*n* control = 1303, *n* intervention = 1420). The mean age was 9.1 years (SD = 0.9), 49.7% were boys, and 62.4% met international PA guidelines at baseline (see Table 2). The proportion meeting PA guidelines was lower in the intervention group (60.5%) than in the control group (64.5%, *P* = 0.032).

**Table 2:** Baseline characteristics of the study sample

	Whole sample				Accelerometer-wearing subsample			
	Total (*n* = 2723)	Control (*n* = 1303)	Intervention (*n* = 1420)	*P *value	Total (*n* = 160)	Control (*n* = 97)	Intervention (*n* = 63)	*P *value
Sociodemographic variables								
Age, mean (SD), years	9.06 (0.86)	9.00 (0.79)	9.12 (0.92)	< 0.001	8.85 (0.79)	8.95 (0.79)	8.71 (0.78)	0.07
Male gender, *n* (%)	1353/2722 (49.71)	648/1302 (49.77)	705 (49.65)	0.95	79 (49.38)	49 (50.52)	30 (47.62)	0.72
Outcome variables								
Meeting international PA guidelines, *n* (%)	1699 (62.39)	840 (64.47)	859 (60.49)	0.032	92 (57.50)	60 (61.86)	32 (50.79)	0.17
Mean PA duration, mean (SD), minutes/day	92.72 (69.78)	95.28 (68.51)	90.37 (70.88)	0.007	76.85 (48.70)	80.38 (49.74)	71.41 (46.92)	0.24
TPB variables								
Attitudes, mean (SD)	3.68 (0.40)	3.70 (0.39)	3.66 (0.41)	0.042	3.69 (0.38)	3.70 (0.39)	3.66 (0.37)	0.44
Subjective norms, mean (SD)	3.16 (0.50)	3.13 (0.50)	3.18 (0.49)	0.017	3.10 (0.56)	3.15 (0.57)	3.03 (0.55)	0.19
PBC, mean (SD)	3.33 (0.58)	3.33 (0.58)	3.33 (0.58)	0.88	3.30 (0.60)	3.35 (0.58)	3.22 (0.62)	0.17
Intentions, mean (SD)	3.37 (0.63)	3.37 (0.63)	3.38 (0.64)	0.52	3.29 (0.68)	3.32 (0.70)	3.24 (0.66)	0.49

*AbbreviationsPA* physical activity, *PBC* perceived behavioral control, *SD* standard deviation, *TPB* theory of planned behavior

Accelerometer-wearing subsample refers to children from the whole sample who were in the subsample planned to wear an accelerometer and who had valid accelerometer data at baseline

Among the children assessed at baseline, 2475 were assessed at 4 months (90.9%; control = 93.7%, intervention = 88.3%), 1974 at 12 months (72.5%; control = 80.3%, intervention = 65.3%), and 1885 at 16 months (69.2%; control = 82%, intervention = 57.5%) (see Fig. 1). The loss-to-follow-up rate at 16 months was significantly higher in the intervention than in the control group and was associated with older age (*Ps* < 0.001) (see Additional file 5). Among the 232 children from the accelerometer-wearing subsample, 160 (69%) had valid data at baseline. Among the 160 children, 87 had valid data at 4 months (54.4%), 91 at 12 months (58.9%), and 78 at 16 months (48.7%) (see Additional file 3). Baseline characteristics of the accelerometer-wearing subsample (*N* = 160) did not differ significantly between the intervention and control groups (*Ps* > 0.05) (see Table 2).

### Preliminary analyses

Internal consistency for all TPB variables at each time point was acceptable (Cronbach’s alpha ≥ 0.63 [83]). All TPB variables were significantly correlated across four-time points (0.19 ≤ *rs* ≤ 0.66, *P*s < 0.001) (see Additional file 6). In terms of magnitude, the majority of correlation coefficients were below the large-effect threshold of 0.50 proposed by Cohen [79]. Based on Funder and Ozer’s guidelines [81], approximately half of all correlations were small-to-moderate in magnitude (*r* < 0.30), while the other half reached a large magnitude (*r* ≥ 0.30). Age (0.08 ≤ *rs* ≤ 0.16, *P*s < 0.001) and gender (0.01 ≤ partial ƞ² ≤ 0.02, *P*s < 0.001) showed small correlations or effect sizes with mean daily PA at each time point [79, 81] (see Additional files 6 and 7). In the accelerometer subsample, self-reported and accelerometry-based PA were significantly correlated at each time point (0.17 ≤ *rs* ≤ 0.25, *Ps* ≤ 0.028), indicating small-to-medium correlations in magnitude [81]. However, Lin’s concordance correlation coefficients indicated low agreement between the two methods (0.08 ≤ *ρc* ≤ 0.16, *Ps* ≤ 0.006) (see Additional file 8).

### Impact of the great live and move challenge on meeting international physical activity guidelines (H_1a_)

**Fig. 3:**
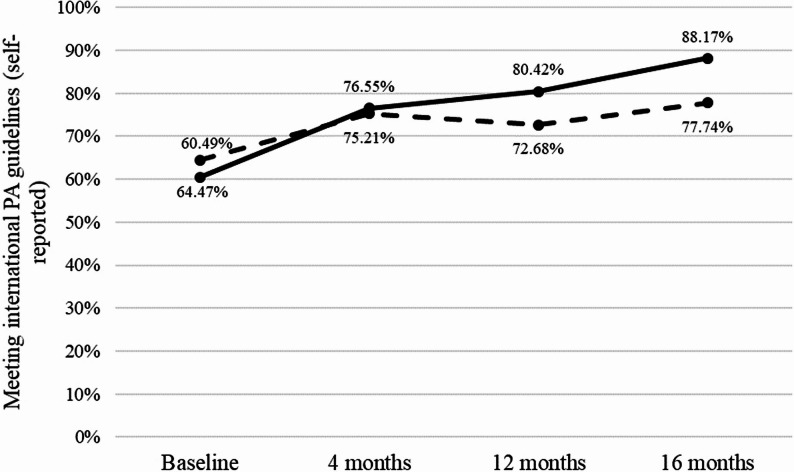
Proportion of children meeting international physical activity guidelines (≥ 60 min/day) at baseline, 4, 12, and 16 months in the intervention and control groups, based on self-reported data Note. Intervention group = solid line; Control group = dashed line. Baseline, pre-intervention of the first school year; 4 months, post-intervention of the first school year; 12 months, pre-intervention of the second school year; 16 months, post-intervention of the second school year. PA = physical activity

The “time x group” interaction indicated that compared to the control group, the intervention group had a significantly higher proportion of children meeting international PA guidelines after 16 months (OR = 3.38, 95% CI = 2.50 to 4.56, *P* < 0.001) (see Table 3; Fig. 3). Accordingly, at 16 months the proportion of children meeting international PA guidelines was 10.4% points higher in the intervention group compared to the control group. In the complete case analysis (*N* = 1680), the “time x group” interaction at 16 months remained significant, also indicating that a higher proportion of children in the intervention group met international PA guidelines after 16 months compared to the control group (OR = 2.76, 95% CI = 1.92 to 3.95, *P* < 0.001) (see Additional file 9). In addition, the complete case analysis that included a random effect for school class, in addition to community of communes, yielded consistent results, also indicating that a higher proportion of children in the intervention group met international PA guidelines after 16 months compared to the control group (OR = 2.91, 95% CI = 1.87 to 4.52, *P* < 0.001; see Additional file 9).

**Table 3:** Changes in the proportion of children meeting international physical activity guidelines according to the randomization group for physical activity

	Control (*n* = 1303)			Intervention (*n* = 1420)			Interaction	
	No. (%) of children	OR [95%CI]	*P* value^a^	No. (%) of children	OR [95% CI]	*P* value^b^	OR [95% CI]	*P* value^c^
Meeting international PA guidelines								
Baseline	840 (64.47)	Ref.		859 (60.49)	Ref.		Ref.	
4 months	980 (75.21)	1.98 [1.63; 2.42]	< 0.001	1087 (76.55)	3.00 [2.46; 3.67]	< 0.001	1.43 [1.08; 1.88]	0.012
12 months	947 (72.68)	1.67 [1.37; 2.03]	< 0.001	1142 (80.42)	4.10 [3.33; 5.06]	< 0.001	2.31 [1.75; 3.06]	< 0.001
16 months	1013 (77.74)	2.39 [1.95; 2.92]	< 0.001	1252 (88.17)	8.79 [6.93; 11.2]	< 0.001	3.38 [2.50; 4.56]	< 0.001

Baseline, pre-intervention of first follow-up year; 4 months, post-intervention of first follow-up year; 12 months, pre-intervention of second follow-up year; 16 months, post-intervention of second follow-up year

*AbbreviationsCI* confidence interval, *OR* odds ratio, *PA* physical activity, *Ref.* reference.

^a^Subgroup analysis (control group) compared with baseline, adjusted for the age of the children, gender of the children, and baseline classification as meeting or not meeting international PA guidelines

^b^Subgroup analysis (intervention group) compared with baseline, adjusted for the age of the children, gender of the children, and baseline classification as meeting or not meeting international PA guidelines

^c^Interaction between time (compared with baseline) and group (intervention group compared to control group), adjusted for the age of the children, gender of the children, and baseline classification as meeting or not meeting international PA guidelines

In the accelerometer-wearing subsample (*N* = 160), based on self-reported data, the “time x group” interaction indicated that compared to the control group, the intervention group had a significantly higher proportion of children meeting international PA guidelines after 16 months (OR = 4.54, 95% CI = 1.34 to 15.40, *P* = 0.015). However, compared to the control group, the intervention group did not show a statistically significant difference in accelerometer-based measures at 16 months (*P* = 0.98; see Additional file 10).

### Impact of the Great Live and Move Challenge on mean daily physical activity and theory of planned behavior variables (H_1b_)

The “time x group” interaction indicated that compared to the control group, the intervention group had significantly higher mean daily PA after 16 months (ES = 0.14, 95% CI = 0.10 to 0.18, *P* < 0.001) (see Table 4). Regarding TPB variables, the “time x group” interactions indicated that compared to the control group, the intervention group had a significantly higher level of attitudes after 16 months (ES = 0.05, 95% CI = 0.01 to 0.08, *P* = 0.016), but significantly lower levels of subjective norms (ES = −0.07, 95% CI = −0.11 to −0.03, *P* < 0.001) and perceived behavioral control (ES = −0.04, 95% CI = −0.08 to −0.01, *P* = 0.043) after 16 months. Results were generally consistent for mean daily PA and TPB variables in the complete case analyses (*N* = 1680) (see Additional file 11). For example, the “time x group” interaction for mean daily PA at 16 months remained significant, thus also indicating higher mean daily PA in the intervention group compared to the control group at 16 months (ES = 0.15, 95% CI = 0.10 to 0.20, *P* < 0.001). A notable exception in the complete case analysis was the absence of significant difference regarding attitudes between the intervention and control groups at 16 months (ES = 0.03, 95% CI = −0.02 to 0.08, *P* = 0.26).

**Table 4:** Changes in mean daily minutes of physical activity and theory of planned behavior variables according to the randomization group

	Control (*n* = 1303)			Intervention (*n* = 1420)			Interaction	
	Mean (SD)	Effect size [95% CI]	*P* value^a^	Mean (SD)	Effect size [95% CI]	*P* value^b^	Effect size [95% CI]	*P* value^c^
Mean PA duration (minutes/day)								
Baseline	95.28 (65.51)	Ref.		90.37 (70.88)	Ref.		Ref.	
4 months	112.88 (83.37)	0.20 [0.14; 0.25]	< 0.001	115.70 (82.84)	0.35 [0.30; 0.41]	< 0.001	0.07 [0.03; 0.11]	< 0.001
12 months	101.67 (65.65)	0.12 [0.06; 0.17]	< 0.001	105.80 (61.02)	0.35 [0.29; 0.40]	< 0.001	0.11 [0.07; 0.15]	< 0.001
16 months	111.31 (66.70)	0.25 [0.20; 0.31]	< 0.001	120.61 (59.88)	0.55 [0.50; 0.61]	< 0.001	0.14 [0.10; 0.18]	< 0.001
Attitudes								
Baseline	3.70 (0.39)	Ref.		3.66 (0.41)	Ref.		Ref.	
4 months	3.70 (0.38)	0.02 [-0.04; 0.07]	0.56	3.72 (0.38)	0.17 [0.11; 0.22]	< 0.001	0.07 [0.03; 0.11]	< 0.001
12 months	3.69 (0.38)	-0.03 [-0.08; 0.03]	0.36	3.70 (0.34)	0.11 [0.06; 0.16]	< 0.001	0.06 [0.03; 0.10]	0.001
16 months	3.69 (0.38)	-0.03 [-0.08; 0.03]	0.35	3.69 (0.33)	0.07 [0.02; 0.12]	0.010	0.05 [0.01; 0.08]	0.016
Subjective norms								
Baseline	3.13 (0.50)	Ref.		3.18 (0.49)	Ref.		Ref.	
4 months	3.27 (0.45)	0.31 [0.25; 0.36]	< 0.001	3.29 (0.43)	0.27 [0.22; 0.33]	< 0.001	-0.03 [-0.07; 0.01]	0.11
12 months	3.27 (0.41)	0.30 [0.25; 0.36]	< 0.001	3.27 (0.36)	0.21 [0.16; 0.26]	< 0.001	-0.06 [-0.10; -0.02]	0.003
16 months	3.29 (0.41)	0.36 [0.30; 0.41]	< 0.001	3.28 (0.33)	0.25 [0.20; 0.30]	< 0.001	-0.07 [-0.11; -0.03]	< 0.001
PBC								
Baseline	3.33 (0.58)	Ref.		3.33 (0.58)	Ref.		Ref.	
4 months	3.42 (0.54)	0.18 [0.12; 0.23]	< 0.001	3.40 (0.56)	0.14 [0.09; 0.20]	< 0.001	-0.02 [-0.06; 0.01]	0.21
12 months	3.41 (0.52)	0.16 [0.10; 0.21]	< 0.001	3.39 (0.50)	0.13 [0.08; 0.18]	< 0.001	-0.02 [-0.06; 0.02]	0.28
16 months	3.44 (0.51)	0.22 [0.16; 0.27]	< 0.001	3.41 (0.46)	0.16 [0.10; 0.21]	< 0.001	-0.04 [-0.08; -0.01]	0.043
Intentions								
Baseline	3.37 (0.63)	Ref.		3.38 (0.64)	Ref.		Ref.	
4 months	3.45 (0.59)	0.13 [0.08; 0.19]	< 0.001	3.45 (0.56)	0.11 [0.06; 0.16]	< 0.001	-0.01 [-0.05; 0.02]	0.46
12 months	3.50 (0.54)	0.22 [0.17; 0.27]	< 0.001	3.48 (0.50)	0.18 [0.12; 0.23]	< 0.001	-0.03 [-0.07; 0.01]	0.15
16 months	3.53 (0.53)	0.27 [0.21; 0.32]	< 0.001	3.52 (0.46)	0.25 [0.20; 0.30]	< 0.001	-0.02 [-0.05; 0.02]	0.37

*AbbreviationsCI* confidence interval, *PA* physical activity, *PBC* perceived behavioral control, *Ref.* reference, *SD* standard deviation

Baseline, pre-intervention of first follow-up year; 4 months, post-intervention of first follow-up year; 12 months, pre-intervention of second follow-up year; 16 months, post-intervention of second follow-up year

^a^Subgroup analysis (control group) compared with baseline, adjusted for the age of the children, gender of the children, and baseline level of the variable (mean daily minutes of PA or score on the theory of planned behavior variable)

^b^Subgroup analysis (intervention group) compared with baseline, adjusted for the age of the children, gender of the children, and baseline level of the variable (mean daily minutes of PA or score on the theory of planned behavior variable)

^c^Interaction between time (compared with baseline) and group (intervention group compared to control group), adjusted for the age of the children, gender of the children, and baseline level of the variable (mean daily minutes of PA or score on the theory of planned behavior variable)

### Path analytic models (H_2a_ and H_2b_)

The model assessing the GLMC’s impact on children’s mean daily PA through TPB variables (H_2a_) showed an acceptable fit with the data (see Table 5). Compared to the control group, the intervention group had a significant direct effect on baseline-4 months changes in attitudes (β = 0.05, 95% CI = 0.02 to 0.09, *P* < 0.001) and mean daily PA at 16 months (β = 0.07, 95% CI = 0.04 to 0.10, *P* < 0.001) (see Table 6). However, compared to the control group, the intervention group did not have a significant indirect effect on the mean daily PA at 16 months (β = 0.01, 95% CI = 0.00 to 0.03, *P* = 0.07) (see Additional file 12). In the complete case analysis for H_2a_ (*N* = 1680), direct effects in the tested model were globally similar to those observed in the main analysis, with the intervention group showing, for example, a significant direct effect on mean daily PA at 16 months compared to the control group (β = 0.09, 95% CI = 0.05 to 0.14, *P* < 0.001) (see Additional file 13).

**Table 5:** Goodness-of-fit indices for the models exploring the impact of the Great Live and Move Challenge through the theory of planned behavior variables and the links between the theory of planned behavior variables according to the randomization group

Model	χ²	df	*P *value	CFI	TLI	RMSEA	SRMR
Impact of the GLMC through TPB variables (H_2a_)							
Total sample	461.60	79	< 0.001	0.96	0.93	0.04	0.06
Links between TPB variables according to the randomization group (H_2b_)							
Control group	246.25	76	< 0.001	0.96	0.94	0.04	0.06
Intervention group	282.35	76	< 0.001	0.96	0.94	0.04	0.07

*AbbreviationsCFI* comparative fit index, *df* degree of freedom, *GLMC* Great Live and Move Challenge, *RMSEA* root mean square error of approximation, *SRMR* standardized root mean square residual, *TLI* Tucker–Lewis index, *TPB* theory of planned behavior, *χ²* chi-square statistic

**Table 6:** Standardized parameter estimates for the direct effects of the path analytic models exploring the impact of the Great Live and Move Challenge through the theory of planned behavior variables and the links between the theory of planned behavior variables according to the randomization group

Independent variable	Dependent variable	Impact of the GLMC through TPB variables (H_2a_)		Links between TPB variables according to the randomization group (H_2b_)				
		Total (n = 2723)		Control (n = 1303)		Intervention (n = 1420)		
		β [95% CI]	P value	β [95% CI]	P value	β [95% CI]	P value	CR^a^
Paths from the randomization group to the basic model variables								
Randomization group	Change in attitudes (Baseline-4 months)	0.05 [0.02, 0.09]	< 0.001					
Randomization group	Change in SN (Baseline-4 months)	0.0 [-0.04, 0.03]	0.87					
Randomization group	Change in PBC (Baseline-4 months)	-0.02 [-0.06, 0.01]	0.23					
Randomization group	Mean daily minutes of PA(4 months)	0.03 [-0.01, 0.06]	0.12					
Randomization group	Mean daily minutes of PA(12 months)	0.04 [0.0, 0.07]	0.028					
Randomization group	Change in attitudes (12-16 months)	-0.02 [-0.06, 0.01]	0.22					
Randomization group	Change in SN (12-16 months)	-0.02 [-0.06, 0.02]	0.34					
Randomization group	Change in PBC (12-16 months)	-0.03 [-0.07, 0.01]	0.10					
Randomization group	Mean daily minutes of PA(16 months)	0.07 [0.04, 0.1]	< 0.001					
Paths for the TPB variables between baseline and 4 months								
Change in attitudes (Baseline-4 months)	Intentions (4 months)	0.16 [0.12, 0.2]	< 0.001	0.17 [0.11, 0.22]	< 0.001	0.16 [0.11, 0.21]	< 0.001	0.29
Change in SN (Baseline-4 months)	Intentions (4 months)	0.16 [0.12, 0.19]	< 0.001	0.16 [0.11, 0.21]	< 0.001	0.15 [0.11, 0.2]	< 0.001	0.50
Change in PBC (Baseline-4 months)	Intentions (4 months)	0.32 [0.28, 0.36]	< 0.001	0.29 [0.23, 0.34]	< 0.001	0.35 [0.30, 0.4]	< 0.001	1.29
Change in PBC (Baseline-4 months)	Mean daily minutes of PA(4 months)	0.09 [0.05, 0.12]	< 0.001	0.13 [0.08, 0.18]	< 0.001	0.04 [-0.03, 0.1]	0.27	2.47†
Intentions (4 months)	Mean daily minutes of PA(4 months)	0.17 [0.13, 0.20]	< 0.001	0.13 [0.08, 0.17]	< 0.001	0.22 [0.16, 0.27]	< 0.001	2.45†
Autoregressive paths								
Intentions (Baseline)	Intentions (4 months)	0.42 [0.39, 0.45]	< 0.001	0.45 [0.40, 0.49]	< 0.001	0.40 [0.35, 0.43]	< 0.001	2.94†
Intentions (4 months)	Intentions (12 months)	0.40 [0.36, 0.44]	< 0.001	0.37 [0.31, 0.43]	< 0.001	0.43 [0.38, 0.48]	< 0.001	1.31
Intentions (12 months)	Intentions (16 months)	0.46 [0.42, 0.50]	< 0.001	0.47 [0.42, 0.53]	0.001	0.45 [0.39, 0.51]	< 0.001	1.73
Intentions (Baseline)	Intentions (12 months)	0.19 [0.15, 0.23]	< 0.001	0.18 [0.12, 0.25]	< 0.001	0.20 [0.15, 0.26]	< 0.001	0.01
Intentions (Baseline)	Intentions (16 months)	0.05 [0.01, 0.08]	0.006	0.05 [0.00, 0.10]	0.032	0.04 [0.00, 0.09]	0.069	0.56
Intentions (4 months)	Intentions (16 months)	0.16 [0.15, 0.20]	< 0.001	0.16 [0.10, 0.21]	< 0.001	0.17 [0.11, 0.22]	< 0.001	0.12
Mean daily minutes of PA (Baseline)	Mean daily minutes of PA(4 months)	0.29 [0.24, 0.33]	< 0.001	0.28 [0.22, 0.34]	< 0.001	0.30 [0.24, 0.36]	< 0.001	0.21
Mean daily minutes of PA (4 months)	Mean daily minutes of PA(12 months)	0.23 [0.19, 0.28]	< 0.001	0.20 [0.14, 0.25]	0.001	0.28 [0.22, 0.34]	< 0.001	1.80
Mean daily minutes of PA (12 months)	Mean daily minutes of PA(16 months)	0.31 [0.27, 0.36]	< 0.001	0.31 [0.24, 0.38]	< 0.001	0.32 [0.25, 0.38]	< 0.001	0.12
Mean daily minutes of PA (Baseline)	Mean daily minutes of PA(12 months)	0.18 [0.14, 0.22]	< 0.001	0.22 [0.16, 0.27]	0.001	0.15 [0.10, 0.21]	< 0.001	2.37†
Mean daily minutes of PA (Baseline)	Mean daily minutes of PA(16 months)	0.16 [0.12, 0.20]	< 0.001	0.18 [0.12, 0.24]	< 0.001	0.14 [0.09, 0.2]	< 0.001	1.66
Mean daily minutes of PA (4 months)	Mean daily minutes of PA(16 months)	0.12 [0.08, 0.15]	0.001	0.11 [0.06, 0.16]	< 0.001	0.12 [0.07, 0.18]	< 0.001	0.04
Change in attitudes (Baseline-4 months)	Change in attitudes (12-16 months)	0.15 [0.11, 0.20]	< 0.001	0.13 [0.07, 0.19]	< 0.001	0.18 [0.12, 0.25]	< 0.001	1.09
Change in SN (Baseline-4 months)	Change in SN (12-16 months)	0.10 [0.06, 0.14]	< 0.001	0.11 [0.04, 0.17]	0.001	0.10 [0.04, 0.15]	< 0.001	0.67
Change in PBC (Baseline-4 months)	Change in PBC (12-16 months)	0.09 [0.04, 0.13]	< 0.001	0.08 [0.02, 0.14]	0.005	0.10 [0.04, 0.16]	0.002	0.05
Paths for the TPB variables between 12 and 16 months								
Change in attitudes (12-16 months)	Intentions (16 months)	0.14 [0.09, 0.18]	< 0.001	0.14 [0.08, 0.20]	< 0.001	0.14 [0.07, 0.2]	< 0.001	0.14
Change in SN (12-16 months)	Intentions (16 months)	0.1 [0.06, 0.14]	< 0.001	0.13 [0.08, 0.18]	< 0.001	0.08 [0.03, 0.14]	0.002	0.92
Change in PBC (12-16 months)	Intentions (16 months)	0.24 [0.19, 0.28]	< 0.001	0.11 [0.06, 0.16]	< 0.001	0.26 [0.19, 0.33]	< 0.001	1.64
Change in PBC (12-16 months)	Mean daily minutes of PA (16 months)	0.06 [0.02, 0.10]	0.003	0.07 [0.02, 0.13]	0.006	0.04 [-0.02, 0.1]	0.17	0.90
Intentions (16 months)	Mean daily minutes of PA (16 months)	0.19 [0.15, 0.22]	< 0.001	0.14 [0.09, 0.19]	< 0.001	0.24 [0.19, 0.29]	< 0.001	2.87†
Paths from sociodemographic variables to mean daily minutes of PA								
Gender	Mean daily minutes of PA (Baseline)	0.10 [0.07, 0.14]	< 0.001	0.12 [0.07, 0.17]	< 0.001	0.09 [0.04, 0.14]	< 0.001	0.68
Gender	Mean daily minutes of PA (4 months)	0.07 [0.04, 0.11]	< 0.001	0.09 [0.04, 0.14]	0.001	0.06 [0.01, 0.11]	0.018	0.89
Gender	Mean daily minutes of PA (12 months)	0.09 [0.06, 0.12]	< 0.001	0.05 [0.00, 0.10]	0.046	0.13 [0.09, 0.18]	< 0.001	2.26†
Gender	Mean daily minutes of PA (16 months)	0.04 [0.01, 0.07]	0.012	0.04 [-0.01, 0.09]	0.11	0.04 [0.00, 0.09]	0.049	0.02
Age	Mean daily minutes of PA (Baseline)	0.14 [0.10, 0.17]	< 0.001	0.14 [0.09, 0.19]	< 0.001	0.14 [0.10, 0.19]	< 0.001	0.10
Age	Mean daily minutes of PA (4 months)	0.04 [0.00, 0.07]	0.023	0.04 [-0.02, 0.08]	0.22	0.04 [-0.01, 0.08]	0.079	0.01
Age	Mean daily minutes of PA (12 months)	0.03 [0.00, 0.06]	0.037	0.00 [-0.05, 0.05]	0.98	0.06 [0.02, 0.1]	0.002	1.70
Age	Mean daily minutes of PA (16 months)	0.01 [-0.02, 0.04]	0.57	-0.01 [-0.05, 0.04]	0.85	0.02 [-0.02, 0.06]	0.30	0.74

*Abbreviationsβ*standardized parameter estimate, *CI*confidence interval, *CR*critical ratio,*GLMC*Great Live and Move Challenge, *PA*physical activity, *PBC*perceived behavioral control, *SN*subjective norms, *TPB* theory of planned behavior

Baseline, pre-intervention of first follow-up year; 4 months, post-intervention of first follow-up year; 12 months, pre-intervention of second follow-up year; 16 months, post-intervention of second follow-up year. Randomization group, allocation to the intervention or control group

^a^Critical ratio value for statistical difference in path coefficient between intervention and control groups

† Significant difference between the path coefficient of the control and intervention groups (critical ratio >1.96)

Regarding analyses exploring whether the link between TPB variables varied according to the randomization group (H_2b_), the model showed an acceptable fit with the data for the control and intervention groups (see Table 5). The path coefficient between intentions at 4 months and mean daily PA at 4 months was significantly higher (CR = 2.45) in the intervention group (β = 0.22, 95% CI = 0.16 to 0.27, *P* < 0.001) compared to the control group (β = 0.13, 95% CI = 0.08 to 0.17, *P* < 0.001) (see Table 6). Similarly, the path coefficient between intentions at 16 months and mean daily PA at 16 months was significantly higher (CR = 2.87) in the intervention group (β = 0.24, 95% CI = 0.19 to 0.29, *P* < 0.001) compared to the control group (β = 0.14, 95% CI = 0.09 to 0.19, *P* < 0.001). In the complete case analysis for H_2b_, results were similar to those observed in the main analysis (see Additional file 13). Notably, the path coefficient between intentions at 4 months and mean daily PA at 4 months was significantly higher (CR = 1.96) in the intervention group (β = 0.23, 95% CI = 0.17 to 0.29, *P* < 0.001) compared to the control group (β = 0.13, 95% CI = 0.07 to 0.19, *P* < 0.001), and the path coefficient between intentions at 16 months and mean daily PA at 16 months was significantly higher (CR = 2.23) in the intervention group (β = 0.24, 95% CI = 0.18 to 0.30, *P* < 0.001) compared to the control group (β = 0.14, 95% CI = 0.08 to 0.20, *P* < 0.001).

## Discussion

The present study indicates that the GLMC effectively increases the proportion of children aged 7–11 years who meet international guidelines of at least 60 min of daily PA. In line with H_1a_, a significantly higher increase was found in the proportion of children meeting the international PA guidelines in the intervention group (27.7%) compared to the control group during follow-up (13.3%), resulting in a 10.4% point difference between groups at the end of the study. At 16 months, 88.2% of children in the intervention group met the PA guidelines. The impact of the GLMC on the proportion of children meeting international PA guidelines at 16 months can be considered a medium effect size based on OR [78], which is particularly encouraging, considering the negligible mean impact of interventions promoting PA in children, as reported by previous meta-analyses [12–15]. It is worth noting, however, that the observed 10.4% difference between intervention and control groups in the proportion of children meeting international PA guidelines was lower than the 15% difference initially hypothesized in H_1a_. One likely explanation is that the proportion of children meeting international PA guidelines at baseline was substantially higher in the present sample (more than 60%) than expected in the protocol (35%) [52]. With a majority of children already meeting international PA guidelines at baseline, there was less room for improvement, which may have limited the magnitude of the between-group difference over the follow-up. Nonetheless, these findings from the present study remain highly informative for schools and public health policymakers, as the GLMC is a brief PA intervention (i.e., six weeks) that can be repeated annually. Moreover, as recommended by the World Health Organization [84], the GLMC involves a broad community of education stakeholders (i.e., teachers, parents, municipal officials, and public policy stakeholders), ensuring children receive the appropriate prompts to be more active in various settings (e.g., home, school, community) [52]. Beyond this supportive environment, the positive impact of the GLMC on the proportion of children meeting PA guidelines may be notably explained by the shared goal of accumulating as many “energy cubes” as possible, which may have fostered a strong sense of belonging to a collective community among both children and educational stakeholders [85]. In addition, the relatively short duration of the GLMC (i.e., six weeks, including four weeks of “volitional phase”) may have helped to prevent declines in children’s engagement with accumulating and counting their “energy cubes,” as disengagement over time in interventions including a self-monitoring BCT has been reported in previous research [86]. Interestingly, beyond the higher increase observed in the intervention group during the first (baseline-4 months) and the second editions (12–16 months) of the GLMC compared to the control group (see Table 3), the proportion of children meeting PA guidelines was sustained and even slightly increased in the intervention group between the two editions (4–12 months, + 3.9%), contrasting with a slight decrease in the control group between the two editions (−2.5%). The combination of the intervention’s brevity and the shared goal of accumulating “energy cubes” may have generated a particularly engaging experience during the first edition of the GLMC, contributing both to the maintenance of PA among children and to sustaining a supportive environment by educational stakeholders between the two editions. Taken as a whole, these results highlight a potential “cumulative” impact of the GLMC on children’s PA from one school year to the next. Future research should explore whether the proportion of children meeting PA guidelines continues to rise with longer GLMC implementation, such as when children benefit from the intervention from the first to fifth grades. Finally, although the increase was consistently higher in the intervention group during both the first (baseline-4 months) and second editions (12–16 months) of the GLMC, the proportion of children meeting PA guidelines in the control group also increased over these periods. Given that across both school years the time points preceding the GLMC were conducted in winter (January-February; baseline and 12 months) and those following the GLMC in late spring (May-June; 4 and 16 months), this increase in the control group is likely attributable to seasonal variation [87]. Indeed, in European countries with a temperate climate, such as France, children’s PA has been found to be lower in winter than in spring [88].

Regarding results from H_1b_, the significant impact of the GLMC was confirmed when PA was treated as a continuous variable in daily minutes, with an ES at 16 months considered small [79]. Analyses related to TPB variables indicated that, after 16 months, the intervention group had a significantly higher level of attitudes (although not confirmed in the complete case analysis) while the control group had a significantly higher level of subjective norms and perceived behavioral control, with all ES falling within the very small or trivial range [79]. Analyses related to H_2a_ indicated that the GLMC’s indirect effect on mean daily PA at 16 months via TPB variables was not significant. Overall, these results indicate that the present research failed to align with a core principle of theory-based interventions, which is to demonstrate that the mechanisms of change proposed by the selected theory significantly mediate the intervention’s impact on behavior [29]. There are at least two potential explanations for these findings. One relates to a ceiling effect as baseline attitudes, norms, perceived behavioral control, and intentions were already high in both groups (Means ≥ 3.13 for control, ≥ 3.18 for intervention, see Table 2), leaving little room for any medium or large impact due to the GLMC [81]. If confirmed by future research, such results would mean that children are already motivated toward PA and that the GLMC “motivational phase” targeting TPB determinants of intentions would not be essential [30]. Another explanation is that the BCTs used in the GLMC may have produced changes through mechanisms beyond the TPB variables measured in the present study. Indeed, the literature highlights some inconsistencies and a lack of consensus about which mechanisms are specifically impacted by certain BCTs [89]. This means that some BCTs could plausibly have influenced unmeasured processes not captured by the TPB. For instance, the “Information about health consequences” BCT in the GLMC to present the advantages of practicing PA regularly may not have impacted attitudes as hypothesized (see Table 1) [23] but did impact other variables not included in the TPB like perceived susceptibility and vulnerability [89]. In addition, Module 5 (“Let’s encourage all children to do PA!”) was designed to target children’s subjective norms through the use of the BCTs “Information about health consequences”, “Information about social and environmental consequences”, and “Information about emotional consequences”, while mobilizing education stakeholders involved in the GLMC (i.e., teachers, parents, municipal officials, and policy makers) to support children’s PA. Beyond subjective norms, this module may therefore have influenced broader environmental and social constructs such as perceived family support [90] or perceived teacher support [91]. To explore whether the program impacts children’s PA via processes beyond TPB, future research could build on existing literature linking BCTs and mechanisms of change [17, 89], and also rely on expert consensus analyses to formally map the BCTs implemented in the GLMC to their most likely mechanisms of change [92].

When investigating whether the link between TPB variables varied by randomization group (H_2b_), results showed a significantly stronger intentions-PA link in the intervention group compared to the control group after each GLMC edition (4 and 16 months). Given the existing intentions-PA gap [31, 32, 41], it has been suggested that TPB-based interventions could be optimized by including a “volitional phase” with contents facilitating the translation of intentions into PA (e.g., PA sessions, implementation intentions) [40, 47]. However, this study is the first to report empirically that an intervention can strengthen the intentions-PA link. Such a result has theoretical and interventional implications. From a theoretical viewpoint, it should be noted that beyond TPB, numerous psychosocial theories, like protection motivation theory [93] or social cognitive theory [94], include intentions as the most proximal antecedent to PA [95]. Including content to facilitate the translation of intentions into PA could benefit interventions based on these theories, as it might enhance their efficacy without challenging intentions as the proximal determinant of PA [42–44]. From an interventional viewpoint, theory-based interventions could more systematically include content reinforcing the links between mechanisms of change, in addition to impacting the levels of mechanisms of change [29, 48]. It should be noted that beyond the intentions-behavior gap, numerous links between mechanisms of change could be strengthened, such as the attitudes-intentions link [96].

Several limitations should be noted. First, parental consent was relatively low (59.82%), resulting in a smaller sample than the 4000 required (2803 [70%] assessed at baseline) [52]. This participation level is consistent with previous research using active parental consent procedures (i.e., requiring parents to return a signed form for their child’s participation) [97]. Nevertheless, the relatively low consent rate may have reduced the representativeness of the achieved sample, as families agreeing for their child to participate could differ from those declining (e.g., higher interest in PA promotion), which may in turn reduce the generalizability of the current findings [98]. Of note, the statistical power remained satisfactory, close to 90%, due to differences between expected and observed parameters used to estimate the sample size (in particular, intra-class correlation coefficient and the average number of participants per community of communes) [99], which were more favorable to a smaller sample size. Second, the intervention group had a significantly higher attrition rate than the control group, and attrition was associated with older age, which, while common in health trials [100, 101], may have reduced the representativeness of the retained sample and thereby threatened the study’s internal validity [102] and limited the generalizability of the findings [98]. The high attrition rate in the intervention group seems mainly due to teachers withdrawing during follow-up (see Fig. 1), although the reasons for withdrawal were not systematically recorded in the study protocol. In school-based intervention research, participant follow-up may depend not only on individual-level factors (e.g., children leaving school), but also on school personnel such as teachers, who for instance decided whether or not to continue to involve their class in the GLMC, or on broader staffing decisions at the school level [103]. The number of teacher withdrawals in the intervention group raises concerns about the GLMC’s acceptability to teachers, which could be investigated through more systematic collection of data on reasons for teacher withdrawal and a process evaluation in the future [104]. Third, a key limitation was the primary reliance on a self-reported method to measure PA. Although PA was assessed using an adapted version of the PAQ-C [66], which has been identified by expert consensus as one of the few self-report instruments with acceptable validity, reliability, and feasibility for use in children [105], self-reported methods measuring PA in children remain subject to various measurement errors including subjectivity, social desirability bias, and variable recall ability [106]. In addition, our adapted version, which asked children to report PA duration in minutes across various contexts (e.g., school recess, lunchtime, daily life activities) and then calculated mean daily PA, may have increased measurement errors and potential overestimation compared to the original PAQ-C scoring [107]. Consistent with these limitations, correlations between self-reported PA (PAQ-C) and accelerometer-based PA (collected from a small subsample, *N* = 160) were modest, providing limited evidence for concurrent validity between the two measurement methods. Exploratory analyses in this subsample did not show a significant GLMC effect on accelerometer‑based proportion of children meeting international PA guidelines, despite a significant effect being observed on self‑reported PA (PAQ-C) in the same children. This discrepancy may be partly explained by the fact that, beyond the limitations of self-reported methods reported above, accelerometer-based measures may be also subject to some measurement limitations (e.g., under-monitoring of certain activities, natural day-to-day variability in movement patterns) [108, 109], which can attenuate the detection of intervention effects [110]. Combined with the small sample size of the accelerometer-wearing subsample in the present study, these potential limitations of accelerometer-based PA may have reduced the statistical power to detect between-group differences. Future studies should confirm the GLMC’s impact on PA using accelerometer data on a large sample. Fourth, although questionnaires were administered in classrooms supervised by the teacher and study assessors, and items were adapted from previous TPB studies and previous studies measuring PA based on the PAQ-C in children to ensure age-appropriate wording for 7–11-year-old respondents (e.g., [22, 66, 74, 75]), the reading ability of some children, notably the youngest participants (7-year-olds), may still have posed challenges. This potential limitation should be considered when interpreting the self-reported data of the present study. Fifth, no measures of intervention fidelity were collected in this trial. Although the GLMC was delivered using standardized role-specific intervention guides and tailored support from the GLMC staff (for teachers, municipal officials, and policy stakeholders), the absence of systematic fidelity data prevents determining whether the intervention was consistently applied as intended across schools and various settings (e.g., extracurricular time, weekend PA sessions in the cities’ neighborhoods). This limitation reinforces need to include a process evaluation to notably assess the fidelity of the GLMC delivery in future research [104].

## Conclusions

The present study indicates that the GLMC, a TPB-based intervention, effectively promoted PA in French primary school children aged 7–11 over 16 months. The intentions-PA link was significantly stronger in the intervention group at 4 and 16 months, suggesting that the GLMC may help bridge the intentions-PA gap [31]. However, there was no evidence that TPB variables mediated the GLMC’s efficacy. Future research should continue to explore the underlying mechanisms involved in the GLMC’s efficacy on PA [29], assess the GLMC’s impact on health indicators (e.g., cardiorespiratory fitness) [111], explore the GLMC’s long-term impact after the intervention has finished [112], and determine the GLMC’s transferability to other contexts, such as other French regions [113].

## Supplementary Information

Supplementary Material 1.

Supplementary Material 2.

Supplementary Material 3.

Supplementary Material 4.

Supplementary Material 5.

Supplementary Material 6.

Supplementary Material 7.

Supplementary Material 8.

Supplementary Material 9.

Supplementary Material 10.

Supplementary Material 11.

Supplementary Material 12.

Supplementary Material 13.

## Data Availability

The datasets used and/or analyzed during the current study are available from the corresponding author on reasonable request.

## Abbreviations

BCT: Behavior change technique
CFI: Comparative fit index
CI: Confidence interval
CONSORT: Consolidated Standards of Reporting Trials
CR: Critical ratio
ES: Effect size
GLMC: Great Live and Move Challenge
ISRCTN: International Standard Randomized Controlled Trial Number
PA: Physical activity
PAQ-C: Physical activity questionnaire for children
RMSEA: Root mean square error of approximation
SD: Standard deviation
SRMR: Standardized root mean square residual
TIDieR: Template for Intervention Description and Replication
TLI: Tucker-Lewis index
TPB: Theory of planned behavior
