# Supplementary material for: The great live and move challenge and the promotion of physical activity in children: results from a two-school-year cluster-randomized trial
Source: Int J Behav Nutr Phys Act. 2025 Dec 1;23:1. doi: 10.1186/s12966-025-01849-x (PMC12781596; doi:10.1186/s12966-025-01849-x)
Supplement: Supplementary file 3 — Supplementary Material 3. [file 12966_2025_1849_MOESM3_ESM.docx]

**Additional file 3.** CONSORT flow diagram of participants in the accelerometer-wearing subsample.

Children subsample from the control group

(*n* = 182)

Children from the control group not randomized to wear an accelerometer (*n* = 61)

Children from the control group in the accelerometer-wearing subsample (*n* = 121)

- With accelerometer valid data (*n* = 97)

**Enrollment**

Children subsample consented to wear an accelerometer

(*n* = 340)

Children consented to take part in the Great Live and Move Challenge study (*n* = 2803)

Excluded (*n* = 2463 children)

- Did not return consent or declined to wear an accelerometer (*n* = 2463)

**Allocation – Baseline**

**4 months**

**12 months**

**16 months**

Children with accelerometer valid data (*n* = 53)

Children with accelerometer valid data (*n* = 55)

Children with accelerometer valid data (*n* = 52)

Children subsample from the intervention group

(*n* = 158)

Children from the intervention group not randomized to wear an accelerometer (*n* = 47)

Children from the intervention group in the accelerometer-wearing subsample (*n* = 111)

- With accelerometer valid data (*n* = 63)

Children with accelerometer valid data (*n* = 53)

Children with accelerometer valid data (*n* = 34)

Children with accelerometer valid data (*n* = 26)

*Note*. Baseline, pre-intervention of first follow-up year; 4 months, post-intervention of first follow-up year; 12 months, pre-intervention of second follow-up year; 16 months, post-intervention of second follow-up year.

All participating children (*N* = 2803) were invited to take part in the accelerometer-based assessment. Parental consent was obtained for 340 children, who constituted the accelerometer-wearing subsample. From this subsample, a random selection was conducted within the intervention and control groups, respectively, to determine which children would wear an accelerometer at each time point.
