# Supplementary material for: The great live and move challenge and the promotion of physical activity in children: results from a two-school-year cluster-randomized trial
Source: Int J Behav Nutr Phys Act. 2025 Dec 1;23:1. doi: 10.1186/s12966-025-01849-x (PMC12781596; doi:10.1186/s12966-025-01849-x)
Supplement: Supplementary file 4 — Supplementary Material 4. [file 12966_2025_1849_MOESM4_ESM.docx]

**Additional file 4.** Detailed presentation of the basic model used to explore the underlying mechanisms of the impact of the Great Live and Move Challenge on children’s mean daily physical activity.

Figure 2 in the manuscript presents the basic model, common to the analyses linked to hypotheses H_2a_ and H_2b_, to explore the underlying mechanisms of the impact of the Great Live and Move Challenge on children’s mean daily minutes of physical activity over the 16-month follow-up. Given the potential number of variables and complexity of this model, changes in mean scores of attitudes, subjective norms, and perceived behavioral control between baseline and 4 months (i.e., first school year) and between 12 and 16 months (i.e., second school year), were estimated using residual change scores, respectively (Castro-Schilo & Grimm, 2018). Residual change scores report the amount of increase or decrease in a given variable over time (i.e., between baseline and 4 months or 12 and 16 months), while taking into account the initial score of the variable (i.e., at baseline or 12 months) (Aelterman et al., 2016). Such an approach is recognized as a useful way to take into account changes in variables over time while minimizing parameterization (Castro-Schilo & Grimm, 2018). Otherwise, changes in intentions and mean daily minutes of physical activity over the four time points were estimated using autoregressive paths (Kenny, 2005). When exploring autoregressive effects, variables are regressed on themselves over time. Such a procedure allows for examining change in a given variable from one time point to another unconfunded with prior relationships with the same variable (Rhodes et al., 2006). Using autoregressive paths for changes in intentions and mean daily minutes of physical activity over the four time points in the basic model allowed for testing specific links between intentions and mean daily minutes of physical activity at 4 and 16 months, respectively, in line with hypothesis H_2b_.

The basic model included paths for the variables of the theory of planned behavior between baseline and 4 months (i.e., baseline-4 months changes in attitudes, subjective norms, and perceived behavioral control link to intentions at 4 months, baseline-4 months change in perceived behavioral control and intentions at 4 months link to mean daily minutes of physical activity at 4 months), the identical sequence for the variables of the theory of planned behavior between 12 and 16 months (i.e., 12-16 months changes in attitudes, subjective norms, and perceived behavioral control link to intentions at 16 months, 12-16 months change in perceived behavioral control and intentions at 16 months link to mean daily minutes of physical activity at 16 months), and autoregressive paths (i.e., links between measures of mean daily minutes of physical activity over the four-time points, links between intentions over the four-time points, links between baseline-4 months changes in attitudes, subjective norms, and perceived behavioral control and 12-16 months changes in attitudes, subjective norms, and perceived behavioral control, respectively). The basic model also included links between gender and age with mean daily minutes of physical activity at the four time points (Schwarzfischer et al., 2019). In addition, the error terms between changes in attitudes, subjective norms, and perceived behavioral control over the baseline-4 months and 12-16 months periods were allowed to covary, respectively (Ajzen, 1985; Hagger et al., 2001). The error terms between intentions at 12 months and mean daily minutes of physical activity at 12 months were also allowed to covary.

**References**

Aelterman, N., Vansteenkiste, M., Van Keer, H., & Haerens, L. (2016). Changing teachers’ beliefs regarding autonomy support and structure: The role of experienced psychological need satisfaction in teacher training. *Psychology of Sport and Exercise*, *23*, 64–72. https://doi.org/10.1016/j.psychsport.2015.10.007

Ajzen, I. (1985). From intentions to action: A theory of planned behavior. In J. Kuhl & J. Beckman (Eds.), *Action control:From cognitions to behaviors* (pp. 11–39). Springer.

Castro-Schilo, L., & Grimm, K. J. (2018). Using residualized change versus difference scores for longitudinal research. *Journal of Social and Personal Relationships*, *35*(1), 32–58. https://doi.org/10.1177/0265407517718387

Hagger, M. S., Chatzisarantis, N., Biddle, S. J. H., & Orbell, S. (2001). Antecedents of children’s physical activity intentions and behaviour: Predictive validity and longitudinal effects. *Psychology & Health*, *16*(4), 391–407.

Kenny, D. A. (2005). Cross‐lagged panel design. In B. S. Everitt & D. C. Howel (Eds.), *Encyclopedia of statistics in behavioral science*. John Wiley & Sons, Ltd. https://doi.org/10.1002/0470013192.bsa156

Rhodes, R. E., Macdonald, H. M., & McKay, H. A. (2006). Predicting physical activity intention and behaviour among children in a longitudinal sample. *Social Science & Medicine*, *62*(12), 3146–3156. https://doi.org/10.1016/j.socscimed.2005.11.051

Schwarzfischer, P., Gruszfeld, D., Stolarczyk, A., Ferre, N., Escribano, J., Rousseaux, D., Moretti, M., Mariani, B., Verduci, E., Koletzko, B., & Grote, V. (2019). Physical activity and sedentary behavior from 6 to 11 years. *Pediatrics*, *143*(1), e20180994. https://doi.org/10.1542/peds.2018-0994
