# Supplementary material for: The great live and move challenge and the promotion of physical activity in children: results from a two-school-year cluster-randomized trial
Source: Int J Behav Nutr Phys Act. 2025 Dec 1;23:1. doi: 10.1186/s12966-025-01849-x (PMC12781596; doi:10.1186/s12966-025-01849-x)
Supplement: Supplementary file 5 — Supplementary Material 5. [file 12966_2025_1849_MOESM5_ESM.docx]

|  | Dropouts  (*n* = 838) | Completers  (*n* = 1885) | *P* value |
| --- | --- | --- | --- |
| Group, *n* (%) |  |  | < 0.001 |
| Control | 235 (28.04) | 1068 (56.66) |  |
| Intervention | 603 (71.96) | 817 (43.34) |  |
| Sociodemographic variables |  |  |  |
| Age, mean (SD), years | 9.39 (0.97) | 8.92 (0.76) | < 0.001 |
| Male gender, *n* (%) | 412/837 (49.22) | 941 (49.92) | 0.74 |
| Outcome variables |  |  |  |
| Meeting international PA guidelines, *n* (%) | 527 (62.89) | 1172 (62.18) | 0.72 |
| Mean PA duration, mean (SD), minutes/day | 98.24 (76.27) | 90.27 (66.57) | 0.07 |
| TPB variables |  |  |  |
| Attitudes, mean (SD) | 3.66 (0.42) | 3.69 (0.39) | 0.12 |
| Subjective norms, mean (SD) | 3.13 (0.50) | 3.17 (0.50) | 0.08 |
| PBC, mean (SD) | 3.30 (0.59) | 3.34 (0.57) | 0.12 |
| Intentions, mean (SD) | 3.36 (0.64) | 3.38 (0.63) | 0.27 |

**Additional file 5.** Comparison of baseline characteristics between study completers (*n* = 1885) and dropouts (*n* = 838).

Abbreviations: PA, physical activity; PBC, perceived behavioral control; SD, standard deviation; TPB, theory of planned behavior.

Note: Comparisons were performed at baseline. Completers were defined as children with available data at 16 months. Dropouts were defined as children lost to follow-up at 16 months.
