# Supplementary material for: The great live and move challenge and the promotion of physical activity in children: results from a two-school-year cluster-randomized trial
Source: Int J Behav Nutr Phys Act. 2025 Dec 1;23:1. doi: 10.1186/s12966-025-01849-x (PMC12781596; doi:10.1186/s12966-025-01849-x)
Supplement: Supplementary file 6 — Supplementary Material 6. [file 12966_2025_1849_MOESM6_ESM.docx]

| Variables | 1 | 2 | 3 | 4 | 5 | 6 | 7 | 8 | 9 | 10 | 11 | 12 | 13 | 14 | 15 | 16 | 17 | 18 | 19 | 20 | 21 |
| --- | --- | --- | --- | --- | --- | --- | --- | --- | --- | --- | --- | --- | --- | --- | --- | --- | --- | --- | --- | --- | --- |
| 1. Age |  |  |  |  |  |  |  |  |  |  |  |  |  |  |  |  |  |  |  |  |  |
| 2. Attitudes (Baseline) | 0.05** |  |  |  |  |  |  |  |  |  |  |  |  |  |  |  |  |  |  |  |  |
| 3. SN (Baseline) | 0.06** | 0.36*** |  |  |  |  |  |  |  |  |  |  |  |  |  |  |  |  |  |  |  |
| 4. PBC (Baseline) | 0.08*** | 0.57*** | 0.41*** |  |  |  |  |  |  |  |  |  |  |  |  |  |  |  |  |  |  |
| 5. Intentions (Baseline) | 0.07*** | 0.48*** | 0.45*** | 0.61*** |  |  |  |  |  |  |  |  |  |  |  |  |  |  |  |  |  |
| 6. Mean daily minutes of PA (Baseline) | 0.13*** | 0.19*** | 0.17*** | 0.26*** | 0.28*** |  |  |  |  |  |  |  |  |  |  |  |  |  |  |  |  |
| 7. Attitudes (4 months) | 0.00 | 0.49*** | 0.26*** | 0.36*** | 0.29*** | 0.16*** |  |  |  |  |  |  |  |  |  |  |  |  |  |  |  |
| 8. SN (4 months) | 0.03 | 0.28*** | 0.53*** | 0.31*** | 0.31*** | 0.15*** | 0.40*** |  |  |  |  |  |  |  |  |  |  |  |  |  |  |
| 9. PBC (4 months) | 0.01 | 0.36*** | 0.26*** | 0.53*** | 0.38*** | 0.22*** | 0.58*** | 0.39*** |  |  |  |  |  |  |  |  |  |  |  |  |  |
| 10. Intentions (4 months) | 0.02 | 0.34*** | 0.32*** | 0.41*** | 0.46*** | 0.24*** | 0.50*** | 0.45*** | 0.61*** |  |  |  |  |  |  |  |  |  |  |  |  |
| 11. Mean daily minutes of PA (4 months) | 0.08*** | 0.15*** | 0.13*** | 0.20*** | 0.20*** | 0.35*** | 0.21*** | 0.18*** | 0.27*** | 0.28*** |  |  |  |  |  |  |  |  |  |  |  |
| 12. Attitudes (12 months) | -0.04* | 0.40*** | 0.21*** | 0.30*** | 0.24*** | 0.13*** | 0.57*** | 0.29*** | 0.40*** | 0.36*** | 0.18*** |  |  |  |  |  |  |  |  |  |  |
| 13. SN (12 months) | 0.03 | 0.22*** | 0.42*** | 0.24*** | 0.23*** | 0.14*** | 0.28*** | 0.56*** | 0.28*** | 0.33*** | 0.16*** | 0.39*** |  |  |  |  |  |  |  |  |  |
| 14. PBC (12 months) | 0.02 | 0.31*** | 0.23*** | 0.47*** | 0.34*** | 0.19*** | 0.41*** | 0.28*** | 0.62*** | 0.45*** | 0.23*** | 0.59*** | 0.39*** |  |  |  |  |  |  |  |  |
| 15. Intentions (12 months) | 0.00 | 0.27*** | 0.27*** | 0.38*** | 0.38*** | 0.21*** | 0.38*** | 0.32*** | 0.47*** | 0.49*** | 0.23*** | 0.52*** | 0.45*** | 0.66*** |  |  |  |  |  |  |  |
| 16. Mean daily minutes of PA (12 months) | 0.12*** | 0.15*** | 0.14*** | 0.23*** | 0.22*** | 0.33*** | 0.21*** | 0.16*** | 0.25*** | 0.25*** | 0.37*** | 0.26*** | 0.21*** | 0.31*** | 0.35*** |  |  |  |  |  |  |
| 17. Attitudes (16 months) | -0.05* | 0.38*** | 0.19*** | 0.31*** | 0.23*** | 0.14*** | 0.55*** | 0.27*** | 0.40*** | 0.32*** | 0.19*** | 0.66*** | 0.30*** | 0.48*** | 0.44*** | 0.21*** |  |  |  |  |  |
| 18. SN (16 months) | 0.01 | 0.21*** | 0.39*** | 0.24*** | 0.23*** | 0.15*** | 0.26*** | 0.50*** | 0.26*** | 0.31*** | 0.17*** | 0.33*** | 0.64*** | 0.34*** | 0.37*** | 0.19*** | 0.38*** |  |  |  |  |
| 19. PBC (16 months) | 0.00 | 0.29*** | 0.22*** | 0.45*** | 0.32*** | 0.19*** | 0.37*** | 0.27*** | 0.56*** | 0.41*** | 0.23*** | 0.45*** | 0.30*** | 0.66*** | 0.54*** | 0.26*** | 0.60*** | 0.41*** |  |  |  |
| 20. Intentions (16 months) | -0.01 | 0.30*** | 0.23*** | 0.35*** | 0.34*** | 0.18*** | 0.35*** | 0.30*** | 0.44*** | 0.46*** | 0.23*** | 0.43*** | 0.35*** | 0.54*** | 0.60*** | 0.28*** | 0.53*** | 0.42*** | 0.66*** |  |  |
| 21. Mean daily minutes of PA (16 months) | 0.09*** | 0.12*** | 0.14*** | 0.21*** | 0.21*** | 0.34*** | 0.20*** | 0.15*** | 0.23*** | 0.25*** | 0.34*** | 0.21*** | 0.17*** | 0.27*** | 0.29*** | 0.47*** | 0.26*** | 0.23*** | 0.32*** | 0.36*** | - |
| Cronbach’s Alpha | - | 0.69 | 0.67 | 0.72 | 0.69 | - | 0.72 | 0.65 | 0.73 | 0.73 | - | 0.76 | 0.63 | 0.77 | 0.75 | - | 0.78 | 0.67 | 0.79 | 0.77 | - |

**Additional file 6.** Reliability statistics and Pearson correlation coefficients between study variables.

Abbreviations: PA, physical activity; PBC, perceived behavioral control; SN, subjective norms.

Note: Baseline, pre-intervention of first follow-up year; 4 months, post-intervention of first follow-up year; 12 months, pre-intervention of second follow-up year; 16 months, post-intervention of second follow-up year. All theory of planned behavior variables were measured on a 4-point scale. **P* < .05; ***P* < .01, ****P* < .001. The correlation coefficients were interpreted as follows (absolute value): Very small or trivial < 0.10, small ≥ 0.10, medium ≥ 0.20, large ≥ 0.30 (Funder & Ozer, 2019).

**References**

Funder, D. C., & Ozer, D. J. (2019). Evaluating effect size in psychological research: Sense and nonsense. *Advances in Methods and Practices in Psychological Science*, *2*(2), 156–168. https://doi.org/10.1177/2515245919847202
