# Supplementary material for: The great live and move challenge and the promotion of physical activity in children: results from a two-school-year cluster-randomized trial
Source: Int J Behav Nutr Phys Act. 2025 Dec 1;23:1. doi: 10.1186/s12966-025-01849-x (PMC12781596; doi:10.1186/s12966-025-01849-x)
Supplement: Supplementary file 7 — Supplementary Material 7. [file 12966_2025_1849_MOESM7_ESM.docx]

**Additional file 7.** MANOVA and ANOVAs examining gender differences between study variables.

|  | | | | | Gender | |
| --- | --- | --- | --- | --- | --- | --- |
|  | ANOVA | | | Girls (*n* = 1369) | | Boys (*n* = 1353) |
| Dependent variable | *F* | *P value* | partial ƞ² | Mean (SD) | | Mean (SD) |
| Attitudes (Baseline) | 0.90 | 0.34 | 0.00 | 3.69 (0.39) | | 3.67 (0.42) |
| SN (Baseline) | 0.74 | 0.45 | 0.00 | 3.17 (0.48) | | 3.15 (0.51) |
| PBC (Baseline) | 7.19 | 0.007 | 0.00 | 3.30 (0.58) | | 3.36 (0.58) |
| Intentions (Baseline) | 8.47 | 0.004 | 0.00 | 3.34 (0.62) | | 3.41 (0.64) |
| Mean daily minutes of PA (Baseline) | 37.85 | < 0.001^a^ | 0.01 | 84.63 (65.65) | | 100.98 (72.82) |
| Attitudes (4 months) | 0.43 | 0.51 | 0.00 | 3.71 (0.38) | | 3.72 (0.39) |
| SN (4 months) | 2.92 | 0.088 | 0.00 | 3.30 (0.42) | | 3.27 (0.46) |
| PBC (4 months) | 9.52 | 0.002^a^ | 0.00 | 3.38 (0.54) | | 3.44 (0.55) |
| Intentions (4 months) | 7.04 | 0.008 | 0.00 | 3.42 (0.56) | | 3.48 (0.58) |
| Mean daily minutes of PA (4 months) | 37.96 | < 0.001^a^ | 0.01 | 104.67 (79.59) | | 124.17 (85.44) |
| Attitudes (12 months) | 1.33 | 0.25 | 0.00 | 3.69 (0.35) | | 3.70 (0.37) |
| SN (12 months) | 2.45 | 0.12 | 0.00 | 3.28 (0.37) | | 3.26 (0.41) |
| PBC (12 months) | 8.38 | 0.004 | 0.00 | 3.37 (0.51) | | 3.43 (0.52) |
| Intentions (12 months) | 12.13 | 0.001^a^ | 0.00 | 3.46 (0.50) | | 3.53 (0.53) |
| Mean daily minutes of PA (12 months) | 66.48 | < 0.001^a^ | 0.02 | 94.11 (59.89) | | 113.66 (65.16) |
| Attitudes (16 months) | 0.14 | 0.71 | 0.00 | 3.68 (0.34) | | 3.69 (0.38) |
| SN (16 months) | 6.60 | 0.010 | 0.00 | 3.31 (0.34) | | 3.27 (0.40) |
| PBC (16 months) | 1.91 | 0.17 | 0.00 | 3.41 (0.47) | | 3.44 (0.50) |
| Intentions (16 months) | 4.96 | 0.026 | 0.00 | 3.51 (0.49) | | 3.55 (0.51) |
| Mean daily minutes of PA (16 months) | 45.44 | < 0.001^a^ | 0.02 | 108.09 (61.85) | | 124.35 (63.93) |

Abbreviations: PA, physical activity; PBC, perceived behavioral control; SD, standard deviation; SN, subjective norms.

Note: Baseline, pre-intervention of first follow-up year; 4 months, post-intervention of first follow-up year; 12 months, pre-intervention of second follow-up year; 16 months, post-intervention of second follow-up year. All theory of planned behavior variables were measured on a 4-point scale. Results for the MANOVA examining gender differences between study variables: *F* (20, 2701) = 7.08, *p* < 0.001, partial ƞ² = 0.05. For ANOVAs effects *df* = 1, 2720. Due to the multiplication of tests, a Bonferroni correction was applied with a significance threshold fixed at 0.05 / 20 = 0.002).

^a^ Significant *P* value after Bonferroni correction (*P* ≤ 0.002).
