# Supplementary material for: The great live and move challenge and the promotion of physical activity in children: results from a two-school-year cluster-randomized trial
Source: Int J Behav Nutr Phys Act. 2025 Dec 1;23:1. doi: 10.1186/s12966-025-01849-x (PMC12781596; doi:10.1186/s12966-025-01849-x)
Supplement: Supplementary file 8 — Supplementary Material 8. [file 12966_2025_1849_MOESM8_ESM.docx]

**Additional file 8.** Results of correlation and concordance analyses between self-reported and accelerometry-based physical activity duration in the accelerometer-wearing subsample (*n* = 160).

| Time point | Spearman’s correlation coefficient [95%CI] | *P* value | Lin’s concordance correlation coefficient [95%CI] | *P* value |
| --- | --- | --- | --- | --- |
| Baseline | 0.25 [0.10; 0.40] | 0.001 | 0.16 [0.07; 0.25] | < 0.001 |
| 4 months | 0.23 [0.08; 0.38] | 0.004 | 0.11 [0.04; 0.18] | 0.002 |
| 12 months | 0.17 [0.02; 0.33] | 0.028 | 0.09 [0.03; 0.16] | 0.006 |
| 16 months | 0.20 [0.06; 0.35] | 0.009 | 0.08 [0.03; 0.13] | 0.001 |

Abbreviations: CI, confidence interval.

Note: Baseline, pre-intervention of first follow-up year; 4 months, post-intervention of first follow-up year; 12 months, pre-intervention of second follow-up year; 16 months, post-intervention of second follow-up year. Physical activity duration is measured in minutes per day.
