# Supplementary material for: The great live and move challenge and the promotion of physical activity in children: results from a two-school-year cluster-randomized trial
Source: Int J Behav Nutr Phys Act. 2025 Dec 1;23:1. doi: 10.1186/s12966-025-01849-x (PMC12781596; doi:10.1186/s12966-025-01849-x)
Supplement: Supplementary file 9 — Supplementary Material 9. [file 12966_2025_1849_MOESM9_ESM.docx]

**Additional file 9.** Sensitivity analyses of changes in the proportion of children meeting international physical activity guidelines on complete cases (*N* = 1680).

|  | Control (*n* = 938) | | |  | Intervention (*n* = 742) | | |  | Interaction | |
| --- | --- | --- | --- | --- | --- | --- | --- | --- | --- | --- |
|  | No. (%) of children | OR [95%CI] | *P* value^a^ |  | No. (%) of children | OR [95%CI] | *P* value^b^ |  | OR [95%CI] | *P* value^c^ |
| Meeting international PA guidelines (self-reported) | | | |  |  |  |  |  |  |  |
| Baseline | 616 (65.67) | Ref. |  |  | 439 (59.16) | Ref. |  |  | Ref. |  |
| 4 months | 691 (73.67) | 1.66 [1.32; 2.09] | < 0.001 |  | 545 (73.45) | 2.59 [1.97; 3.39] | < 0.001 |  | 1.47 [1.04; 2.08] | 0.031 |
| 12 months | 633 (67.48) | 1.12 [0.89; 1.39] | 0.34 |  | 538 (71.16) | 2.20 [1.68; 2.87] | < 0.001 |  | 1.89 [1.34; 2.67] | < 0.001 |
| 16 months | 688 (73.35) | 1.63 [1.29; 2.04] | < 0.001 |  | 604 (81.40) | 4.87 [3.63; 6.52] | < 0.001 |  | 2.76 [1.92; 3.95] | < 0.001 |
| Meeting international PA guidelines (self-reported – including an additional random effect for school class) | | | | | | | | | |  |
| Baseline | 616 (65.7) | Ref. |  |  | 439 (59.2) | Ref. |  |  | Ref. |  |
| 4 months | 691 (73.7) | 1.76 [1.38; 2.25] | < 0.001 |  | 545 (73.5) | 2.84 [2.12; 3.81] | < 0.001 |  | 1.52 [1.05; 2.19] | 0.026 |
| 12 months | 633 (67.5) | 1.08 [0.83; 1.42] | 0.57 |  | 538 (71.2) | 2.18 [1.56; 3.04] | < 0.001 |  | 1.96 [1.28; 2.99] | 0.002 |
| 16 months | 688 (73.3) | 1.59 [1.21; 2.10] | 0.001 |  | 604 (81.4) | 4.94 [3.44; 7.09] | < 0.001 |  | 2.91 [1.87; 4.52] | < 0.001 |

Abbreviations: CI, confidence interval; OR, odds ratio; PA, physical activity; Ref., reference.

Note: Analyses were conducted on complete cases, defined as children with available data at baseline, 4, 12, and 16 months. Baseline, pre-intervention of first follow-up year; 4 months, post-intervention of first follow-up year; 12 months, pre-intervention of second follow-up year; 16 months, post-intervention of second follow-up year.

^a^Subgroup analysis (control group) compared with baseline, adjusted for the age of the children, gender of the children, and baseline classification as meeting or not meeting international PA guidelines.

^b^Subgroup analysis (intervention group) compared with baseline, adjusted for the age of the children, gender of the children, and baseline classification as meeting or not meeting international PA guidelines.

^c^Interaction between time (compared with baseline) and group (intervention group compared to control group), adjusted for the age of the children, gender of the children, and baseline classification as meeting or not meeting international PA guidelines.
