# Supplementary material for: The great live and move challenge and the promotion of physical activity in children: results from a two-school-year cluster-randomized trial
Source: Int J Behav Nutr Phys Act. 2025 Dec 1;23:1. doi: 10.1186/s12966-025-01849-x (PMC12781596; doi:10.1186/s12966-025-01849-x)
Supplement: Supplementary file 10 — Supplementary Material 10. [file 12966_2025_1849_MOESM10_ESM.docx]

**Additional file 10.** Changes in the proportion of children meeting international physical activity guidelines according to the randomization group for self-reported and accelerometry-based physical activity in the accelerometer-wearing subsample (*N* = 160).

|  | Control (*n* = 97) | | |  | Intervention (*n* = 63) | | |  | Interaction | |
| --- | --- | --- | --- | --- | --- | --- | --- | --- | --- | --- |
|  | No. (%) of children | OR [95% CI] | *P* value^a^ |  | No. (%) of children | OR [95% CI] | *P* value^b^ |  | OR [95% CI] | *P* value^c^ |
| Meeting international PA guidelines (self-reported) | | | | | | | | | | |
| Baseline | 60 (61.86) | Ref. |  |  | 32 (50.79) | Ref. |  |  | Ref. |  |
| 4 months | 77 (79.38) | 3.01 [1.45; 6.22] | 0.003 |  | 46 (73.02) | 3.36 [1.44; 7.83] | 0.005 |  | 1.14 [0.38; 3.42] | 0.81 |
| 12 months | 67 (69.07) | 1.52 [0.77; 3.00] | 0.23 |  | 50 (79.37) | 5.09 [2.09; 12.4] | < 0.001 |  | 3.39 [1.13; 10.2] | 0.029 |
| 16 months | 75 (77.32) | 2.59 [1.27; 5.29] | 0.009 |  | 56 (88.89) | 11.5 [4.14; 32.2] | < 0.001 |  | 4.54 [1.34; 15.4] | 0.015 |
| Meeting international PA guidelines (accelerometry) | | | | | | | | | | |
| Baseline | 69 (71.13) | Ref. |  |  | 39 (61.90) | Ref. |  |  | Ref. |  |
| 4 months | 85 (87.63) | 6.66 [2.32; 19.1] | < 0.001 |  | 55 (87.30) | 25.4 [5.07; 128] | < 0.001 |  | 2.23 [0.43; 11.5] | 0.34 |
| 12 months | 63 (64.95) | 0.57 [0.24; 1.35] | 0.20 |  | 38 (60.32) | 0.84 [0.27; 2.67] | 0.77 |  | 1.57 [0.39; 6.35] | 0.53 |
| 16 months | 72 (74.23) | 1.36 [0.56; 3.30] | 0.50 |  | 45 (65.08) | 1.42 [0.44; 4.57] | 0.76 |  | 0.98 [0.24; 4.11] | 0.98 |

Abbreviations: CI, confidence interval; OR, odds ratio; PA, physical activity; Ref., reference.

Note: Baseline, pre-intervention of first follow-up year; 4 months, post-intervention of first follow-up year; 12 months, pre-intervention of second follow-up year; 16 months, post-intervention of second follow-up year.

^a^Subgroup analysis (control group) compared with baseline, adjusted for the age of the children, gender of the children, and baseline classification as meeting or not meeting international PA guidelines.

^b^Subgroup analysis (intervention group) compared with baseline, adjusted for the age of the children, gender of the children, and baseline classification as meeting or not meeting international PA guidelines.

^c^Interaction between time (compared with baseline) and group (intervention group compared with control group), adjusted for the age of the children, gender of the children, and baseline classification as meeting or not meeting international PA guidelines.
