# Supplementary material for: The great live and move challenge and the promotion of physical activity in children: results from a two-school-year cluster-randomized trial
Source: Int J Behav Nutr Phys Act. 2025 Dec 1;23:1. doi: 10.1186/s12966-025-01849-x (PMC12781596; doi:10.1186/s12966-025-01849-x)
Supplement: Supplementary file 11 — Supplementary Material 11. [file 12966_2025_1849_MOESM11_ESM.docx]

**Additional file 11.** Sensitivity analyses of changes in mean daily minutes of physical activity and theory of planned behavior variables on complete cases (*N* = 1680).

|  | Control (*n* = 938) | | |  | Intervention (*n* = 742) | | |  | Interaction | |
| --- | --- | --- | --- | --- | --- | --- | --- | --- | --- | --- |
|  | Mean (SD) | Effect size  [95% CI] | *P* value^a^ |  | Mean (SD) | Effect size  [95% CI] | *P* value^b^ |  | Effect size  [95% CI] | *P* value^c^ |
| Mean PA duration (minutes/day) |  |  |  |  |  |  |  |  |  |  |
| Baseline | 95.15 (67.68) | Ref. |  |  | 84.64 (64.23) | Ref. |  |  | Ref. |  |
| 4 months | 112.72 (84.02) | 0.17 [0.11; 0.24] | < 0.001 |  | 114.09 (85.47) | 0.30 [0.23; 0.37] | < 0.001 |  | 0.06 [0.02; 0.11] | 0.009 |
| 12 months | 102.12 (72.62) | 0.07 [0.002; 0.13] | 0.041 |  | 105.45 (72.61) | 0.29 [0.22; 0.36] | < 0.001 |  | 0.11 [0.06; 0.16] | < 0.001 |
| 16 months | 110.59 (72.65) | 0.19 [0.13; 0.26] | < 0.001 |  | 122.71 (73.31) | 0.49 [0.42; 0.57] | < 0.001 |  | 0.15 [0.10; 0.20] | < 0.001 |
| Attitudes |  |  |  |  |  |  |  |  |  |  |
| Baseline | 3.72 (0.37) | Ref. |  |  | 3.68 (0.39) | Ref. |  |  | Ref. |  |
| 4 months | 3.72 (0.38) | 0.02 [-0.04; 0.09] | 0.50 |  | 3.74 (0.39) | 0.13 [0.06; 0.20] | < 0.001 |  | 0.05 [0.004; 0.10] | 0.033 |
| 12 months | 3.70 (0.40) | -0.03 [-0.09; 0.03] | 0.36 |  | 3.71 (0.39) | 0.07 [0.00; 0.14] | 0.051 |  | 0.05 [0.002; 0.10] | 0.039 |
| 16 months | 3.71 (0.40) | -0.02 [-0.09; 0.04] | 0.46 |  | 3.70 (0.39) | 0.03 [-0.04; 0.10] | 0.40 |  | 0.03 [-0.02; 0.08] | 0.26 |
| Subjective norms |  |  |  |  |  |  |  |  |  |  |
| Baseline | 3.17 (0.49) | Ref. |  |  | 3.20 (0.48) | Ref. |  |  | Ref. |  |
| 4 months | 3.29 (0.46) | 0.27 [0.20; 0.33] | < 0.001 |  | 3.30 (0.44) | 0.22 [0.15; 0.30] | < 0.001 |  | -0.02 [-0.07; 0.02] | 0.33 |
| 12 months | 3.29 (0.44) | 0.27 [0.21; 0.34] | < 0.001 |  | 3.27 (0.41) | 0.14 [0.07; 0.22] | < 0.001 |  | -0.07 [-0.12; -0.02] | 0.005 |
| 16 months | 3.32 (0.43) | 0.32 [0.26; 0.39] | < 0.001 |  | 3.28 (0.40) | 0.18 [0.10; 0.25] | < 0.001 |  | -0.08 [-0.12; -0.03] | 0.002 |
| PBC |  |  |  |  |  |  |  |  |  |  |
| Baseline | 3.36 (0.55) | Ref. |  |  | 3.35 (0.56) | Ref. |  |  | Ref. |  |
| 4 months | 3.45 (0.53) | 0.15 [0.09; 0.22] | < 0.001 |  | 3.40 (0.59) | 0.08 [0.01; 0.15] | 0.031 |  | -0.04 [-0.08; 0.01] | 0.14 |
| 12 months | 3.44 (0.55) | 0.14 [0.08; 0.21] | < 0.001 |  | 3.39 (0.58) | 0.07 [-0.003; 0.14] | 0.06 |  | -0.04 [-0.08; 0.01] | 0.15 |
| 16 months | 3.47 (0.54) | 0.19 [0.13; 0.26] | < 0.001 |  | 3.40 (0.55) | 0.08 [0.01; 0.15] | 0.028 |  | -0.06 [-0.10; -0.01] | 0.023 |
| Intentions |  |  |  |  |  |  |  |  |  |  |
| Baseline | 3.40 (0.61) | Ref. |  |  | 3.40 (0.63) | Ref. |  |  | Ref. |  |
| 4 months | 3.47 (0.59) | 0.11 [0.05; 0.18] | 0.001 |  | 3.44 (0.59) | 0.06 [-0.01; 0.13] | 0.10 |  | -0.02 [-0.07; 0.02] | 0.34 |
| 12 months | 3.52 (0.58) | 0.19 [0.13; 0.26] | < 0.001 |  | 3.47 (0.59) | 0.12 [0.05; 0.19] | 0.001 |  | -0.03 [-0.08; 0.01] | 0.16 |
| 16 months | 3.55 (0.56) | 0.25 [0.18; 0.31] | < 0.001 |  | 3.53 (0.57) | 0.20 [0.13; 0.28] | < 0.001 |  | -0.02 [-0.06; 0.03] | 0.52 |

Abbreviations: CI, confidence interval; PA, physical activity; PBC, perceived behavioral control; Ref., reference; SD, standard deviation.

Note: Analyses were conducted on complete cases, defined as children with available data at baseline, 4, 12, and 16 months. Baseline, pre-intervention of first follow-up year; 4 months, post-intervention of first follow-up year; 12 months, pre-intervention of second follow-up year; 16 months, post-intervention of second follow-up year.

^a^Subgroup analysis (control group) compared with baseline, adjusted for the age of the children, gender of the children, and baseline level of the variable (mean daily minutes of PA or score on the theory of planned behavior variable).

^b^Subgroup analysis (intervention group) compared with baseline, adjusted for the age of the children, gender of the children, and baseline level of the variable (mean daily minutes of PA or score on the theory of planned behavior variable).

^c^Interaction between time (compared with baseline) and group (intervention group compared to control group), adjusted for the age of the children, gender of the children, and baseline level of the variable (mean daily minutes of PA or score on the theory of planned behavior variable).
