# Supplementary material for: The great live and move challenge and the promotion of physical activity in children: results from a two-school-year cluster-randomized trial
Source: Int J Behav Nutr Phys Act. 2025 Dec 1;23:1. doi: 10.1186/s12966-025-01849-x (PMC12781596; doi:10.1186/s12966-025-01849-x)
Supplement: Supplementary file 12 — Supplementary Material 12. [file 12966_2025_1849_MOESM12_ESM.docx]

**Additional file 12.** Standardized parameter estimates for the indirect and total effects of the path analytic model exploring the impact of the Great Live and Move Challenge through the theory of planned behavior variables.

| Independent variable | Dependent variable | Mediator(s) | β [95% CI] | *P* value |
| --- | --- | --- | --- | --- |
| Indirect effects |  |  |  |  |
| Randomization group | Intentions (4 months) | Change in attitudes (Baseline-4 months) Change in SN (Baseline-4 months)  Change in PBC (Baseline-4 months) | 0.00 [-0.02; 0.02] | 0.94 |
| Randomization group | Mean daily minutes of PA (4 months) | Change in attitudes (Baseline-4 months)  Intentions (4 months)  Change in SN (Baseline-4 months)  Change in PBC (Baseline-4 months) | 0.00 [0.01; 0.00] | 0.55 |
| Randomization group | Intentions (12 months) | Change in attitudes (Baseline-4 months) Change in SN (Baseline-4 months)  Change in PBC (Baseline-4 months)  Intentions (4 months) | 0.00 [-0.01; 0.01] | 0.94 |
| Randomization group | Mean daily minutes of PA (12 months) | Change in attitudes (Baseline-4 months) Change in SN (Baseline-4 months)  Change in PBC (Baseline-4 months)  Intentions (4 months)  Mean daily minutes of PA (4 months) | 0.01 [0.00; 0.02] | 0.15 |
| Randomization group | Change in attitudes (12-16 months) | Change in attitudes (Baseline-4 months) | 0.01 [0.00; 0.01] | <0.001 |
| Randomization group | Change in SN (12-16 months) | Change in SN (Baseline-4 months) | 0.00 [0.00; 0.01] | 0.85 |
| Randomization group | Change in PBC (12-16 months) | Change in PBC (Baseline-4 months) | 0.00 [-0.01; 0.00] | 0.17 |
| Change in attitudes (Baseline-4 months) | Mean daily minutes of PA (4 months) | Intentions (4 months) | 0.03 [0.02; 0.04] | <0.001 |
| Change in attitudes (Baseline-4 months) | Intentions (12 months) | Intentions (4 months) | 0.06 [0.05; 0.08] | <0.001 |
| Change in SN (Baseline-4 months) | Mean daily minutes of PA (4 months) | Intentions (4 months) | 0.03 [0.02; 0.04] | <0.001 |
| Change in SN (Baseline-4 months) | Intentions (12 months) | Intentions (4 months) | 0.06 [0.05; 0.08] | <0.001 |
| Change in PBC (Baseline-4 months) | Mean daily minutes of PA (4 months) | Intentions (4 months) | 0.05 [0.04; 0.07] | <.0001 |
| Change in PBC (Baseline-4 months) | Intentions (12 months) | Intentions (4 months) | 0.13 [0.11; 0.15] | <0.001 |
| Mean daily minutes of PA (Baseline) | Mean daily minutes of PA (12 months) | Mean daily minutes of PA (4 months) | 0.07 [0.05; 0.09] | <0.001 |
| Mean daily minutes of PA (Baseline) | Mean daily minutes of PA (16 months) | Mean daily minutes of PA (4 months)  Mean daily minutes of PA (12 months) | 0.11 [0.09; 0.13] | <0.001 |
| Intentions (Baseline) | Intentions (12 months) | Intentions (4 months) | 0.16 [0.14; 0.18] | <0.001 |
| Intentions (Baseline) | Intentions (16 months) | Intentions (4 months)  Intentions (12 months) | 0.23 [0.21; 0.26] | <0.001 |
| Intentions (Baseline) | Mean daily minutes of PA (4 months) | Intentions (4 months) | 0.07 [0.05; 0.09] | <0.001 |
| Intentions (Baseline) | Mean daily minutes of PA (12 months) | Intentions (4 months)  Mean daily minutes of PA (4 months) | 0.02 [0.01; 0.02] | <0.001 |
| Intentions (4 months) | Intentions (16 months) | Intentions (12 months) | 0.18 [0.16; 0.21] | <0.001 |
| Intentions (4 months) | Mean daily minutes of PA (12 months) | Mean daily minutes of PA (4 months) | 0.04 [0.03; 0.06] | <0.001 |
| Mean daily minutes of PA (4 months) | Mean daily minutes of PA (16 months) | Mean daily minutes of PA (12 months) | 0.07 [0.06; 0.09] | <0.001 |
| Change in attitudes (12-16 months) | Mean daily minutes of PA (16 months) | Intentions (16 months) | 0.03 [0.02; 0.03] | <0.001 |
| Change in SN (12-16 months) | Mean daily minutes of PA (16 months) | Intentions (16 months) | 0.02 [0.02; 0.03] | <0.001 |
| Change in PBC (12-16 months) | Mean daily minutes of PA (16 months) | Intentions (16 months) | 0.07 [0.06; 0.08] | <0.001 |
| Gender | Mean daily minutes of PA (12 months) | Mean daily minutes of PA (4 months) | 0.02 [0.01; 0.03] | <0.001 |
| Gender | Mean daily minutes of PA (16 months) | Mean daily minutes of PA (4 months)  Mean daily minutes of PA (12 months) | 0.04 [0.03; 0.06] | <0.001 |
| Age | Mean daily minutes of PA (12 months) | Mean daily minutes of PA (4 months) | 0.01 [0.00; 0.02] | 0.023 |
| Age | Mean daily minutes of PA (16 months) | Mean daily minutes of PA (4 months)  Mean daily minutes of PA (12 months) | 0.02 [0.01; 0.03] | 0.003 |
| Sums of indirect effects |  |  |  |  |
| Randomization group | Intentions (16 months) | Multiple^a^ | -0.01 [-0.03; 0.00] | 0.16 |
| Randomization group | Mean daily minutes of PA (16 months) | Multiple^a^ | 0.01 [0.00; 0.03] | 0.07 |
| Change in attitudes (Baseline-4 months) | Mean daily minutes of PA (12 months) | Multiple^a^ | 0.02 [0.02; 0.03] | <0.001 |
| Change in attitudes (Baseline-4 months) | Intentions (16 months) | Multiple^a^ | 0.08 [0.06; 0.10] | <0.001 |
| Change in attitudes (Baseline-4 months) | Mean daily minutes of PA (16 months) | Multiple^a^ | 0.03 [0.02; 0.03] | <0.001 |
| Change in SN (Baseline-4 months) | Mean daily minutes of PA (12 months) | Multiple^a^ | 0.02 [0.02; 0.03] | <0.001 |
| Change in SN (Baseline-4 months) | Intentions (16 months) | Multiple^a^ | 0.07 [0.05; 0.08] | <0.001 |
| Change in SN (Baseline-4 months) | Mean daily minutes of PA (16 months) | Multiple^a^ | 0.02 [0.02; 0.03] | <0.001 |
| Change in PBC (Baseline-4 months) | Mean daily minutes of PA (12 months) | Multiple^a^ | 0.07 [0.05; 0.08] | <0.001 |
| Change in PBC (Baseline-4 months) | Intentions (16 months) | Multiple^a^ | 0.08 [0.06; 0.09] | <0.001 |
| Change in PBC (Baseline-4 months) | Mean daily minutes of PA (16 months) | Multiple^a^ | 0.07 [0.06; 0.08] | <0.001 |
| Intentions (Baseline) | Mean daily minutes of PA (16 months) | Multiple^a^ | 0.07 [0.05; 0.08] | <0.001 |
| Intentions (4 months) | Mean daily minutes of PA (16 months) | Multiple^a^ | 0.10 [0.08; 0.11] | <0.001 |
| Intentions (12 months) | Mean daily minutes of PA (16 months) | Multiple^a^ | 0.09 [0.07; 0.10] | <0.001 |
| Total effects |  |  |  |  |
| Randomization group | Mean daily minutes of PA (4 months) | Change in attitudes (Baseline-4 months) Change in SN (Baseline-4 months)  Change in PBC (Baseline-4 months  Intentions (4 months)^b^ | 0.03 [-0.01; 0.06] | 0.16 |
| Randomization group | Mean daily minutes of PA (12 months) | Change in attitudes (Baseline-4 months) Change in SN (Baseline-4 months)  Change in PBC (Baseline-4 months  Intentions (4 months)  Mean daily minutes of PA (4 months)^b^ | 0.05 [0.01; 0.08] | 0.015 |
| Randomization group | Mean daily minutes of PA (16 months) | Multiple^b^ | 0.08 [0.05; 0.12] | <0.001 |
| Change in PBC (Baseline-4 months) | Mean daily minutes of PA (4 months) | Intentions (4 months)^b^ | 0.14 [0.10; 0.17] | <0.001 |
| Change in PBC (12-16 months) | Mean daily minutes of PA (16 months) | Intentions (16 months)^b^ | 0.10 [0.07; 0.14] | <0.001 |
| Intentions (Baseline) | Intentions (12 months) | Intentions (4 months)^b^ | 0.36 [0.33; 0.40] | <0.001 |
| Intentions (Baseline) | Intentions (16 months) | Intentions (4 months)  Intentions (12 months)^b^ | 0.28 [0.25; 0.32] | <0.001 |
| Mean daily minutes of PA (Baseline) | Mean daily minutes of PA (12 months) | Mean daily minutes of PA (4 months)^b^ | 0.25 [0.21; 0.29] | <0.001 |
| Mean daily minutes of PA (Baseline) | Mean daily minutes of PA (16 months) | Mean daily minutes of PA (4 months)  Mean daily minutes of PA (12 months)^b^ | 0.27 [0.23; 0.31] | <0.001 |

Abbreviations: β, standardized parameter estimate; CI, confidence interval; SN, subjective norms; PA, physical activity; PBC, perceived behavioral control.

Note: Baseline, pre-intervention of first follow-up year; 4 months, post-intervention of first follow-up year; 12 months, pre-intervention of second follow-up year; 16 months, post-intervention of second follow-up year. Randomization group, allocation to the intervention or control group.

^a^Mediators for this effect included effects of the independent variable on the dependent variable through multiple mediators.

^b^Mediator(s) for this effect included effect(s) of the independent variable on the dependent variable through (multiple) mediator(s) along with the direct effect of the independent variable on the dependent variable.
