# Supplementary material for: The great live and move challenge and the promotion of physical activity in children: results from a two-school-year cluster-randomized trial
Source: Int J Behav Nutr Phys Act. 2025 Dec 1;23:1. doi: 10.1186/s12966-025-01849-x (PMC12781596; doi:10.1186/s12966-025-01849-x)
Supplement: Supplementary file 13 — Supplementary Material 13. [file 12966_2025_1849_MOESM13_ESM.docx]

**Additional file 13.** Complete case analysis (*N* = 1680) for standardized parameter estimates of the direct effects in the path analytic models exploring the impact of the Great Live and Move Challenge through theory of planned behavior variables, and the links between theory of planned behavior variables according to the randomization group.

| Independent variable | Dependent variable | Impact of the GLMC through TPB variables (H_2a_) | | Links between TPB variables according to the randomization group (H_2b_) | | | | |
| --- | --- | --- | --- | --- | --- | --- | --- | --- |
|  |  | Total (n = 1680) |  | Control (n = 938) | | Intervention (n = 742) | |  |
|  |  | β [95% CI] | P value | β [95% CI] | P value | β [95% CI] | P value | CR^a^ |
| Paths from the randomization group to the basic model variables | | | | | | | | |
| Randomization group | Change in attitudes (Baseline-4 months) | 0.04 [-0.01, 0.08] | 0.12 |  |  |  |  |  |
| Randomization group | Change in SN (Baseline-4 months) | 0.00 [-0.05, 0.04] | 0.83 |  |  |  |  |  |
| Randomization group | Change in PBC (Baseline-4 months) | -0.05 [-0.10, 0.0] | 0.057 |  |  |  |  |  |
| Randomization group | Mean daily minutes of PA (4 months) | 0.04 [-0.01, 0.08] | 0.13 |  |  |  |  |  |
| Randomization group | Mean daily minutes of PA (12 months) | 0.04 [0.0, 0.08] | 0.07 |  |  |  |  |  |
| Randomization group | Change in attitudes (12-16 months) | -0.03 [-0.08, 0.02] | 0.24 |  |  |  |  |  |
| Randomization group | Change in SN (12-16 months) | -0.03 [-0.08, 0.02] | 0.25 |  |  |  |  |  |
| Randomization group | Change in PBC (12-16 months) | -0.04 [-0.09, 0.0] | 0.07 |  |  |  |  |  |
| Randomization group | Mean daily minutes of PA (16 months) | 0.09 [0.05, 0.14] | < 0.001 |  |  |  |  |  |
| Paths for the TPB variables between baseline and 4 months | | | | | | | | |
| Change in attitudes (Baseline-4 months) | Intentions (4 months) | 0.18 [0.14, 0.23] | < 0.001 | 0.19 [0.12, 0.25] | < 0.001 | 0.18 [0.11, 0.25] | < 0.001 | 0.26 |
| Change in SN (Baseline-4 months) | Intentions (4 months) | 0.17 [0.13, 0.21] | < 0.001 | 0.15 [0.09, 0.21] | < 0.001 | 0.20 [0.14, 0.26] | < 0.001 | 1.48 |
| Change in PBC (Baseline-4 months) | Intentions (4 months) | 0.33 [0.28, 0.37] | < 0.001 | 0.30 [0.23, 0.35] | < 0.001 | 0.36 [0.30, 0.43] | < 0.001 | -0.72 |
| Change in PBC (Baseline-4 months) | Mean daily minutes of PA (4 months) | 0.09 [0.04, 0.13] | 0.001 | 0.12 [0.06, 0.17] | < 0.001 | 0.05 [-0.03, 0.12] | 0.27 | 1.55 |
| Intentions (4 months) | Mean daily minutes of PA (4 months) | 0.17 [0.13, 0.22] | < 0.001 | 0.13 [0.07, 0.19] | < 0.001 | 0.23 [0.17, 0.29] | < 0.001 | 1.96† |
| Autoregressive paths |  |  |  |  |  |  |  |  |
| Intentions (Baseline) | Intentions (4 months) | 0.40 [0.36, 0.44] | < 0.001 | 0.43 [0.38, 0.48] | < 0.001 | 0.37 [0.31, 0.42] | < 0.001 | 2.04† |
| Intentions (4 months) | Intentions (12 months) | 0.36 [0.31, 0.41] | < 0.001 | 0.33 [0.26, 0.40] | < 0.001 | 0.39 [0.31, 0.45] | < 0.001 | 1.31 |
| Intentions (12 months) | Intentions (16 months) | 0.43 [0.38, 0.47] | < 0.001 | 0.44 [0.38, 0.50] | < 0.001 | 0.40 [0.33, 0.47] | < 0.001 | 1.14 |
| Intentions (Baseline) | Intentions (12 months) | 0.15 [0.10, 0.20] | < 0.001 | 0.16 [0.08, 0.23] | < 0.001 | 0.15 [0.07, 0.22] | < 0.001 | 0.29 |
| Intentions (Baseline) | Intentions (16 months) | 0.05 [0.01, 0.09] | 0.026 | 0.06 [0.0, 0.11] | 0.038 | 0.04 [-0.03, 0.10] | 0.28 | 0.58 |
| Intentions (4 months) | Intentions (16 months) | 0.15 [0.11, 0.20] | < 0.001 | 0.16 [0.10, 0.22] | < 0.001 | 0.14 [0.07, 0.21] | < 0.001 | 0.39 |
| Mean daily minutes of PA (Baseline) | Mean daily minutes of PA (4 months) | 0.26 [0.21, 0.31] | < 0.001 | 0.28 [0.22, 0.34] | < 0.001 | 0.24 [0.16, 0.33] | < 0.001 | 0.34 |
| Mean daily minutes of PA (4 months) | Mean daily minutes of PA (12 months) | 0.23 [0.18, 0.28] | < 0.001 | 0.20 [0.14, 0.26] | 0.001 | 0.27 [0.18, 0.36] | < 0.001 | 1.36 |
| Mean daily minutes of PA (12 months) | Mean daily minutes of PA (16 months) | 0.29 [0.24, 0.34] | < 0.001 | 0.29 [0.23, 0.37] | < 0.001 | 0.29 [0.22, 0.36] | < 0.001 | 0.03 |
| Mean daily minutes of PA (Baseline) | Mean daily minutes of PA (12 months) | 0.18 [0.13, 0.24] | < 0.001 | 0.23 [0.16, 0.30] | < 0.001 | 0.12 [0.04, 0.20] | 0.001 | 2.06† |
| Mean daily minutes of PA (Baseline) | Mean daily minutes of PA (16 months) | 0.15 [0.10, 0.20] | < 0.001 | 0.17 [0.10, 0.24] | < 0.001 | 0.13 [0.05, 0.21] | 0.001 | 0.65 |
| Mean daily minutes of PA (4 months) | Mean daily minutes of PA (16 months) | 0.09 [0.04, 0.14] | 0.021 | 0.09 [0.03, 0.15] | 0.003 | 0.09 [0.01, 0.17] | 0.018 | 0.13 |
| Change in attitudes (Baseline-4 months) | Change in attitudes (12-16 months) | 0.15 [0.09, 0.21] | < 0.001 | 0.12 [0.05, 0.19] | 0.002 | 0.19 [0.10, 0.28] | < 0.001 | 1.58 |
| Change in SN (Baseline-4 months) | Change in SN (12-16 months) | 0.12 [0.06, 0.17] | < 0.001 | 0.13 [0.05, 0.20] | 0.001 | 0.11 [0.03, 0.18] | 0.005 | 0.4 |
| Change in PBC (Baseline-4 months) | Change in PBC (12-16 months) | 0.10 [0.05, 0.15] | < 0.001 | 0.11 [0.05, 0.18] | 0.001 | 0.09 [0.02, 0.17] | 0.016 | 0.77 |
| Paths for the TPB variables between 12 and 16 months | | | | | | | | |
| Change in attitudes (12-16 months) | Intentions (16 months) | 0.15 [0.09, 0.20] | < 0.001 | 0.14 [0.07, 0.20] | < 0.001 | 0.16 [0.08, 0.23] | < 0.001 | 0.34 |
| Change in SN (12-16 months) | Intentions (16 months) | 0.11 [0.07, 0.15] | < 0.001 | 0.13 [0.08, 0.19] | < 0.001 | 0.09 [0.03, 0.15] | 0.003 | 0.94 |
| Change in PBC (12-16 months) | Intentions (16 months) | 0.24 [0.19, 0.30] | < 0.001 | 0.23 [0.15, 0.29] | < 0.001 | 0.27 [0.19, 0.35] | < 0.001 | 1.07 |
| Change in PBC (12-16 months) | Mean daily minutes of PA  (16 months) | 0.06 [0.02, 0.10] | 0.010 | 0.07 [0.01, 0.13] | 0.016 | 0.04 [-0.03, 0.10] | 0.29 | 0.84 |
| Intentions (16 months) | Mean daily minutes of PA  (16 months) | 0.18 [0.14, 0.22] | < 0.001 | 0.14 [0.08, 0.20] | < 0.001 | 0.24 [0.18, 0.30] | < 0.001 | 2.23† |
| Paths from sociodemographic variables to mean daily minutes of PA | | | | | | | | |
| Gender | Mean daily minutes of PA (Baseline) | 0.10 [0.06, 0.15] | < 0.001 | 0.10 [0.04, 0.16] | 0.002 | 0.11 [0.03, 0.18] | 0.006 | 0 |
| Gender | Mean daily minutes of PA  (4 months) | 0.08 [0.04, 0.13] | 0.001 | 0.09 [0.03, 0.15] | 0.002 | 0.07 [0.0, 0.14] | 0.047 | 0.52 |
| Gender | Mean daily minutes of PA  (12 months) | 0.08 [0.04, 0.13] | 0.001 | 0.03 [-0.02, 0.09] | 0.26 | 0.15 [0.08, 0.21] | < 0.001 | 2.5† |
| Gender | Mean daily minutes of PA  (16 months) | 0.04 [0.0, 0.08] | 0.07 | -0.02 [-0.08,0.03] | 0.43 | 0.04 [-0.03, 0.10] | 0.26 | 0.12 |
| Age | Mean daily minutes of PA (Baseline) | 0.13 [0.08, 0.17] | < 0.001 | 0.12 [0.07, 0.18] | < 0.001 | 0.13 [0.06, 0.19] | < 0.001 | 0.1 |
| Age | Mean daily minutes of PA  (4 months) | 0.02 [-0.02, 0.07] | 0.36 | 0.02 [-0.04, 0.08] | 0.54 | 0.02 [-0.04, 0.09] | 0.46 | 0.11 |
| Age | Mean daily minutes of PA  (12 months) | 0.03 [-0.01, 0.08] | 0.18 | -0.01 [-0.07,0.05] | 0.66 | 0.09 [0.02, 0.15] | 0.010 | 2.22† |
| Age | Mean daily minutes of PA  (16 months) | 0.01 [-0.03, 0.05] | 0.75 | -0.02 [-0.08, 0.03] | 0.43 | 0.04 [-0.02, 0.10] | 0.18 | 1.49 |

Abbreviations: β, standardized parameter estimate; CI, confidence interval; CR, critical ratio; χ², chi-square; GLMC, Great Live and Move Challenge; PA, physical activity; PBC, perceived behavioral control; SN, subjective norms; TPB, theory of planned behavior; CFI, comparative fit index; TLI, Tucker–Lewis index; RMSEA, root mean square error of approximation; SRMR, standardized root mean square residual.

Note: Baseline, pre-intervention of first follow-up year; 4 months, post-intervention of first follow-up year; 12 months, pre-intervention of second follow-up year; 16 months, post-intervention of second follow-up year. Randomization group, allocation to the intervention or control group.

^a^ Critical ratio value for statistical difference in path coefficient between intervention and control groups.

† Significant difference between the path coefficient of the control and intervention groups (critical ratio > 1.96).

Model fit indices: For the model testing the impact of the GLMC through TPB variables (H_2a_): χ²(81) = 432.26, *P* < 0.001; CFI = 0.93; TLI = 0.90; RMSEA = 0.05; SRMR = 0.07. For the model testing the links between TPB variables according to the randomization group (H_2b_) - control group: χ²(77) = 265.50, *P* < 0.001; CFI = 0.93; TLI = 0.90; RMSEA = 0.05; SRMR = 0.07. For the model testing the links between TPB variables according to the randomization group (H_2b_) - intervention group: χ²(77) = 216.81, *P* < 0.001; CFI = 0.94; TLI = 0.91; RMSEA = 0.05; SRMR = 0.07.
